# Supplementary material for: Systemwide energy return on investment in a sustainable transition towards net zero power systems
Source: Nat Commun. 2024 Jan 3;15:208. doi: 10.1038/s41467-023-44232-9 (PMC10764355; doi:10.1038/s41467-023-44232-9)
Supplement: Supplementary file 1 — Supplementary Information [file 41467_2023_44232_MOESM1_ESM.pdf]

Supplementary Information

Systemwide energy return on investment in a sustainable transition towards net zero power systems

Hasret Sahin[1*], A.A. Solomon[1], Arman Aghahosseini[1], Christian Breyer[1]

[1] School of Energy Systems, LUT University, Yliopistonkatu 34, Lappeenranta, 53850, Finland

Correspondence to: hasret.sahin@lut.fi, solomon.asfaw@lut.fi

Contents

Supplementary Note 1 9

1 LUT Energy System Transition Model 9

1.1 Underlying concept and its architecture 9

1.2 Technologies........ 11

Supplementary Note 2 12

2 Cumulative Energy Demand Database for LUT-EROI........ 12

2.1 Selection of the system model 12

2.2 Identification of system boundary 13

2.3 Cumulative energy demand as an indicator........ 15

2.4 Applying same energy quality by converting all primary energy category to electricity........ 15

2.5 Unit conversion factors........ 16

2.6 Overcoming the challenges of CED value calculations 16

Supplementary Note 3 18

3 Cumulative Energy Demand Analysis on Technology Level 18

3.1 Renewable energy power plants 18

3.1.1 Photovoltaic (PV) power plants........ 18

3.1.2 Wind power plants........ 20

3.1.3 Hydropower plants 21

3.1.4 Geothermal power plant 21

3.1.5 Wave energy converter........ 21

3.1.6 Bioenergy related power plants 22

3.1.7 Waste-to-energy CHP plant........ 24

3.1.8 Methane CHP plant 25

3.1.9 Fuel cell CHP plant........ 26

3.1.10 Concentrated solar thermal power (CSP) plant 26

3.1.11 Steam turbine (ST) component........ 28

3.2 Fossil-fuelled power plant 28

3.2.1 Coal based power plants........ 28

3.2.2 Gas based power plants 29

3.2.3 Internal combustion engine (ICE) power plants........ 30

3.2.4 Oil CHP plant 31

3.3 Nuclear power plants........ 31

3.4 Short-term and medium-term storage systems 33

3.4.1 Short-term storage systems........ 33

3.4.2 Medium-term storage systems........ 35

3.5 Power-to-methane (e-methane) technologies 36

3.5.1 Electrolysis unit 36

3.5.2 DAC unit 37
3.5.3 Methanation unit 39
3.5.4 Gas storage units (for CH_4 and H_2) 39
3.6 Fuel CED estimations 40
Supplementary Note 4 43
4 LUT-EROI Results 43
4.1 Global analysis 43
4.2 Regional analysis 48
Supplementary Note 5 57
5 Inventory for Specific Technologies 57
Supplementary Note 6 68
6 Annual Energy Investment Flows in the Energy Transition 68
References 71

List of Figures

Supplementary Fig. 1. The modelling process of LUT-ESTM.. 9
Supplementary Fig. 2. The schematic representation of technologies included in LUT-ESTM. The diagram is in an analogy of [4] and is a modified version of [1]. At the bottom, the commodities are shown. Abbreviations: PP, power plant, CHP, combined heat and power plant, ICE, internal combustion engine, GT, gas turbines, CCS, carbon capture and storage, ST, steam turbines, A-CAES, adiabatic compressed air storage, PHES, pumped hydro energy storage, PtX, power-to-X, CSP, concentrated solar thermal power, PtH, power-to-heat, TES, thermal energy storage, AC, alternating current, HVAC, high voltage alternating current, HVDC, high voltage direct current, FT, Fischer-Tropsch................. 10
Supplementary Fig. 3. The system boundary for invested energy and systemwide EROI estimation for LUT-ESTM. A-CAES, adiabatic compressed air storage, CCS, carbon capture and storage, CED, cumulative energy demand, CHP, combined heat and power plant, CSP, concentrated solar thermal power, DAC, direct air capture, GT, gas turbines, ICE, internal combustion engine, LCI, life cycle inventory, PHES, pumped hydro energy storage, PP, power plant, ST, steam turbines, TES DH &DH, thermal energy storage high temperature & district heating.. 14
Supplementary Fig. 4. The methodology workflow diagram of the LUT-EROI model. CED, cumulative energy demand, CSP, Concentrated solar thermal power, EROI, energy return on investment, LCI, life cycle inventory, LR, learning rates, PV, photovoltaic. 17
Supplementary Fig. 5. Power capacities for Global region during the energy transition period. The panels a, b, c, d, e, f show LUT scenarios, f and g show IEA scenarios, and h and i show Teske/DLR scenarios. CCGT, combined-cycle gas turbines, CCS, carbon capture and storage, CHP, combined heat and power plant, CSP, concentrated solar thermal power, ICE, internal combustion engine, OCGT, open-cycle gas turbines, PP, power plant, ST, steam turbines. 43
Supplementary Fig. 6. Storage capacities for Global region during energy transition. The panels a, b, c, d, e, f show LUT scenarios, f and g show IEA scenarios, and h and i show Teske/DLR scenarios. A-CAES, adiabatic compressed air storage, PHES, pumped hydro energy storage, TES DH &DH, thermal energy storage high temperature & district heating. 44
Supplementary Fig. 7. Storage throughput for Global region during energy transition. The panels a, b, c, d, e, f show LUT scenarios, f and g show IEA scenarios, and h and i show Teske/DLR scenarios. A-CAES, adiabatic compressed air storage, PHES, pumped hydro energy storage, TES DH &DH, thermal energy storage high temperature & district heating. 45
Supplementary Fig. 8. Variable renewable energy (VRE) penetration changes in the Global region. VRE penetration relationships between storage energy capacity, curtailment and total loss are shown in the panels a,b, and c. The scenarios are represented as blue lines for BPS scenarios, grey lines for BPS Plus scenarios, yellow lines for IEA scenarios and finally green lines for Teske/DLR scenarios. Note that VRE in this study refers to variable renewable energy, and it only include solar photovoltaics and wind power technologies. Renewable energy and versus are abbreviated as RE and vs, respectively.. 46
Supplementary Fig. 9. Systemwide EROI trends for Global region.The scenarios are represented as blue lines for BPS scenarios, grey lines for BPS Plus scenarios, yellow lines for IEA scenarios and finally green lines for Teske/DLR scenarios. The cut-off point for (G) EROI represents the net electricity fed to the grid (excluding transmission and distribution (T&D) losses) and for (F) EROI is the net electricity delivered to end-users (including T&D losses)......... 47
Supplementary Fig. 10. Systemwide EROI trends for Europe region. The scenarios are represented as blue lines for BPS scenarios, grey lines for BPS Plus scenarios, yellow lines for IEA scenarios and finally green lines for Teske/DLR scenarios. The cut-off point for (G) EROI represents the net electricity fed to the grid (excluding transmission and distribution (T&D) losses) and for (F) EROI is the net electricity delivered to end-users (including T&D losses)......... 48
Supplementary Fig. 11. Systemwide EROI trends for Middle East and North Africa region. The scenarios are represented as blue lines for BPS scenarios, grey lines for BPS Plus scenarios, yellow lines for IEA scenarios and finally green lines for Teske/DLR scenarios. The cut-off point for (G) EROI represents the net electricity fed to the grid (excluding transmission and distribution (T&D) losses) and for (F) EROI is the net electricity delivered to end-users (including T&D losses). 49
Supplementary Fig. 12. Systemwide EROI trends for Eurasia region. The scenarios are represented as blue lines for BPS scenarios, grey lines for BPS Plus scenarios, yellow lines for IEA scenarios and finally green lines for Teske/DLR scenarios. The cut-off point for (G) EROI represents the net electricity fed to the grid (excluding transmission and distribution (T&D) losses) and for (F) EROI is the net electricity delivered to end-users (including T&D losses)......... 50
Supplementary Fig. 13. Systemwide EROI trends for Northeast Asia region. The scenarios are represented as blue lines for BPS scenarios, grey lines for BPS Plus scenarios, yellow lines for IEA scenarios and finally green lines for Teske/DLR scenarios. The cut-off point for (G) EROI represents the net electricity fed to the grid (excluding transmission and distribution (T&D) losses) and for (F) EROI is the net electricity delivered to end-users (including T&D losses)......... 51
Supplementary Fig. 14. Systemwide EROI trends for Southeast Asia region. The scenarios are represented as blue lines for BPS scenarios, grey lines for BPS Plus scenarios, yellow lines for IEA scenarios and finally green lines for Teske/DLR

scenarios. The cut-off point for (G) EROI represents the net electricity fed to the grid (excluding transmission and distribution (T&D) losses) and for (F) EROI is the net electricity delivered to end-users (including T&D losses). 52
Supplementary Fig. 15. Systemwide EROI trends for South Asian Association for Regional Cooperation region. The scenarios are represented as blue lines for BPS scenarios, grey lines for BPS Plus scenarios, yellow lines for IEA scenarios and finally green lines for Teske/DLR scenarios. The cut-off point for (G) EROI represents the net electricity fed to the grid (excluding transmission and distribution (T&D) losses) and for (F) EROI is the net electricity delivered to end-users (including T&D losses).. 53
Supplementary Fig. 16. Systemwide EROI trends for Sub-Saharan Africa region. The scenarios are represented as blue lines for BPS scenarios, grey lines for BPS Plus scenarios, yellow lines for IEA scenarios and finally green lines for Teske/DLR scenarios. The cut-off point for (G) EROI represents the net electricity fed to the grid (excluding transmission and distribution (T&D) losses) and for (F) EROI is the net electricity delivered to end-users (including T&D losses). . 54
Supplementary Fig. 17. Systemwide EROI trends for South America region. The scenarios are represented as blue lines for BPS scenarios, grey lines for BPS Plus scenarios, yellow lines for IEA scenarios and finally green lines for Teske/DLR scenarios. The cut-off point for (G) EROI represents the net electricity fed to the grid (excluding transmission and distribution (T&D) losses) and for (F) EROI is the net electricity delivered to end-users (including T&D losses). 55
Supplementary Fig. 18. Systemwide EROI trends for North America region. The scenarios are represented as blue lines for BPS scenarios, grey lines for BPS Plus scenarios, yellow lines for IEA scenarios and finally green lines for Teske/DLR scenarios. The cut-off point for (G) EROI represents the net electricity fed to the grid (excluding transmission and distribution (T&D) losses) and for (F) EROI is the net electricity delivered to end-users (including T&D losses). 56
Supplementary Fig. 19. Systemwide energy investment flow for the global power system. The panels a, b, c, d, e, f show LUT scenarios, f and g show IEA scenarios, and h and i show Teske/DLR scenarios. EI refers to energy investments, and the annual EI for operation does not include structural materials. FEC represents the final energy consumption for the respective year. 69
Supplementary Fig. 20. Annual energy investment flow for the global power system. The panels a, b, c, d, e, f show LUT scenarios, f and g show IEA scenarios, and h and i show Teske/DLR scenarios. EI refers to energy investments, and the annual EI for operation does not include structural materials. FEC represents the final energy consumption for the respective year. 70

List of Tables

Supplementary Table 1. Primary to electricity conversion factors relevant to ecoinvent database[32–35]. 15
Supplementary Table 2. CED values of PV plants in units of MJ_{el}. 18
Supplementary Table 3. Reference PV CED values according to years. 19
Supplementary Table 4. The global annual production shares of PV module technologies from 2015 to 2050. 20
Supplementary Table 5. CED values of wind power plants in units of MJ_{el}. 20
Supplementary Table 6. CED values of hydropower plants in units of MJ_{el}. 21
Supplementary Table 7. CED values of geothermal power plant in units of MJ_{el}. 21
Supplementary Table 8. CED values of wave energy converter in units of MJ_{el}. 22
Supplementary Table 9. CED values of bioenergy related power plants in units of MJ_{el}. 24
Supplementary Table 10. CED values of WtE CHP in units of MJ_{el}. 25
Supplementary Table 11. CED values of methane CHP plant in units of MJ_{el}. 26
Supplementary Table 12. CED values of fuel cell CHP plant in units of MJ_{el} 26
Supplementary Table 13. CED values of CSP ST in units of MJ_{el}. 27
Supplementary Table 14. The cumulative capacity shares of CSP plant technologies from 2015 to 2050. 27
Supplementary Table 15. Adjusted CSP CED values according to years in units of MJ_{el}. 28
Supplementary Table 16. CED values of ST in units of MJ_{el}. 28
Supplementary Table 17. CED values of coal-based power plants in units of MJ_{el}. 29
Supplementary Table 18. CED values of gas-based power plants in units of MJ_{el}. 30
Supplementary Table 19. CED values of ICE and multifuel ICE in units of MJ_{el}. 30
Supplementary Table 20. CED values of oil CHP plant in units of MJ_{el}. 31
Supplementary Table 21. CED values of nuclear power plants in units of MJ_{el}. 32
Supplementary Table 22. The cumulative capacity shares of nuclear power plant technologies from 2015 to 2050. 32
Supplementary Table 23. Adjusted nuclear power plant CED values according to years in units of MJ_{el}. 32
Supplementary Table 24. CED values of lithium-ion battery in units of MJ_{el}. 34
Supplementary Table 25. Adjusted battery CED value according to years in units of MJ_{el}. 35
Supplementary Table 26. CED values of PHES in units of MJ_{el}. 35
Supplementary Table 27. CED values of A-CAES in units of MJ_{el}. 36
Supplementary Table 28. CED values of TES in units of MJ_{el}. 36
Supplementary Table 29. CED values of electrolysis system in units of MJ_{el}. 37
Supplementary Table 30. Adjusted alkaline electrolysis system CED value according to years in units of MJ_{el}. 37
Supplementary Table 31. CED values of DAC system in units of MJ_{el}. 38
Supplementary Table 32. CED values of methanation system in units of MJ_{el}. 39
Supplementary Table 33. CED values of gas storage system in units of MJ_{el}. 40
Supplementary Table 34. Fuel CED values estimated using Sgouridis et al.[30] in units of MJ_{el}. 41
Supplementary Table 35. Natural gas and oil fuel CED values estimated based Delannoy et al.[107] in units of MJ_{el}. 42
Supplementary Table 36. Life cycle inventory of biomass CHP plant. 57
Supplementary Table 37. Life cycle inventory of CCS unit. 60
Supplementary Table 38. Life cycle inventory of nuclear power plants. 61
Supplementary Table 39. LCI for operation CED value for PWR nuclear power plant. 63
Supplementary Table 40. Life cycle inventory of electrolysis unit components. 64
Supplementary Table 41. LCI for estimating fuel CED value for nuclear power plant. 66

Abbreviations

**AC**	Alternating current
**A-CAES**	Adiabatic compressed air storage
**AEF**	Annualised EROI
**AHWR**	Advanced heavy-water reactor
**APOS**	Allocation at the point of substitution
**a-Si**	Amorphous silicon
**BWR**	Boiling water reactor
**CAGR**	Compound annual growth rate
**CAAGR**	Compound average annual growth rate
**CA-QC**	Canada, Québec
**C-CAES**	Conventional compressed air energy storage
**CCGT**	Combined-cycle gas turbines
**CdTe**	Cadmium telluride
**CED**	Cumulative energy demand
**CH**	Switzerland
**CH_4**	Methane
**CHP**	Combined heat and power
**CIS**	Copper indium selenide
**CO_2**	Carbon dioxide
**c-Si**	Crystalline silicon
**CSP**	Concentrated solar thermal power
**DAC**	Direct air capture
**EI**	Energy investment
**EN**	European standards
**EROI**	Energy return of investment
**FEC**	Final energy consumption
**FBR**	Fast neutron reactor
**GLO**	Global level
**GT**	Gas turbines
**GT-MHR**	Gas turbine modular reactor
**GW**	Gigawatt
**GWh_{th}**	Gigawatt-hour (thermal);
**H_2**	Hydrogen
**HVAC**	High voltage alternating current
**HVDC**	High voltage direct current
**ICE**	Internal combustion engine
**ISO**	International organisation for standardisation
**LCA**	Life cycle assessment
**LCI**	Life cycle inventory
**LHV**	Lower heating value
**LIB**	Lithium-ion batteries
**LR**	Learning rate
**LUT-ESTM**	LUT Energy System Transition Model
**MEA**	Monoethanolamine
**Mono-Si**	Monocrystalline silicon
**MSW**	Municipal solid waste
**NEEDS**	New energy externalities development for sustainability
**NPP**	Nuclear power plant
**OCGT**	Open-cycle gas turbines
**PEI**	Polyethyleneimine
**PEM**	Polymer electrolyte membrane
**PHES**	Pumped hydro energy storage
**PP**	Power plant
**PtG**	Power-to-gas
**PtH**	Power-to-heat
**PtL**	Power-to-liquid
**PtSNG**	Power-to-synthetic natural gas
**PtX**	Power-to-X
**PWR**	Pressurised water reactor
**RER**	European level
**RoW**	Rest of the world
**RU**	Russia
**SMR**	Small modular reactor
**ST**	Steam turbine
**TES**	Thermal energy storage
**TRL**	Technology readiness level
**UK**	United Kingdom
**WtE**	Waste-to-energy

Units

**°C**	Celsius
**g**	Gram
**h**	Hour
**kg**	Kilogram
**km**	Kilometre
**kt**	Kiloton
**kW**	Kilowatt
**kW_{el}**	Kilowatt-electricity
**kWh**	Kilowatt-hour
**kWp**	Kilowatt-peak
**m^3**	Cubic meter
**MJ**	Megajoule
**MW**	Megawatt

Scenarios

**BPS**	Best Policy Scenario
**BPS-NWF**	Best Policy Scenario No Wind Force
**BPS-WF**	Best Policy Scenario Wind Force
**BPS-plus2030**	Best Policy Scenario – Energy Transition completed in 2030
**BPS-plus2035**	Best Policy Scenario – Energy Transition completed in 2035
**BPS-plus2040**	Best Policy Scenario – Energy Transition completed in 2040
**IEA-SDS**	International Energy Agency Sustainable Development Scenario
**IEA-STEPS**	International Energy Agency Stated Policy Scenario
**Teske/DLR-1.5ºC**	Limiting GHG emissions to 1.5ºC targets- not taking into account any political and societal barriers
**Teske/DLR- 2.0ºC**	Limiting GHG emissions to 2.0ºC targets- taking into account any political and societal barriers

Supplementary Note 1

1 LUT Energy System Transition Model

1.1 Underlying concept and its architecture

As a linear optimisation tool, LUT Energy System Transition Model (LUT-ESTM) has a sophisticated architecture, based on a multi-node approach and handling full hourly resolution for an entire year. Its architecture enables the reproduction of the spatial distribution of the sub-regions' demands and supply profiles. Hourly electricity demands of each sub-region are met by its electricity generation considering the restrictions on the available renewable energy sources. The model structure allows for expansion of the power transmission lines capacities in line with the future needs while providing flexibility to the interconnection between the sub-regions. The model's optimisation algorithm aims to reach the minimum system cost providing diversification in various technology types. The total installed capacities of each technology, the associated operating expenses, and relative ramping costs are optimised[1–4].

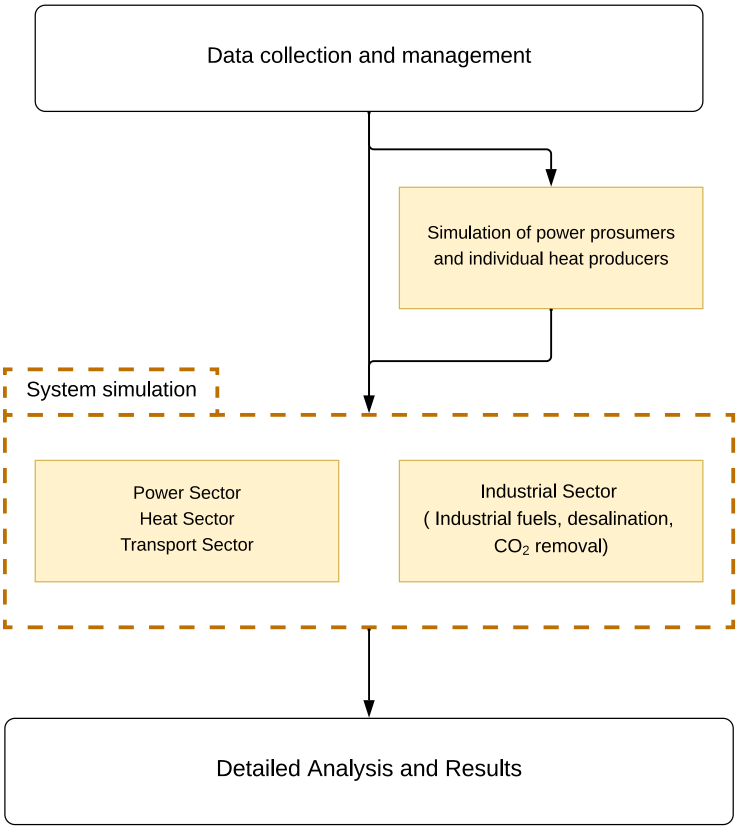

Supplementary Fig. 1. The modelling process of LUT-ESTM.

The modelling process of LUT-ESTM is divided into three main stages: Data preparation, system simulation, and obtaining and evaluation of the results[1]. Technical and financial assumptions are determined according to the current state of existing technologies (commercially available in the global market) and to the technology readiness levels (TRLs) of the new technologies. The transition period of the model is set from

2015 to 2050 with 5-year time intervals[5] to foresee the short-term impacts of technology types in the electricity generation portfolio during the energy transition period. All required data are read from input files and embedded in the model (Supplementary Fig. 1). The optimisation is performed using the MOSEK solver, and the optimisation results are organised in terms of installed capacities, annual electricity generation, cost of system and components, cost of electricity, and CO_2 emissions[6].

LUT-ESTM is a technology-oriented bottom-up model, comprising about 120 technologies. The wide-range of technologies ensure sector-by-sector technology diversity while the associated working architecture allows for flexibility in the configuration of the system to see the commodity exchanges between the sectors. The sectors are structured considering the defined commodities exchanges, mainly electricity, hydrogen, and liquid hydrocarbon, which supports the system continuity and sustainability. LUT-ESTM infrastructure and exchange of commodities are given in Suplementary Fig. 2.

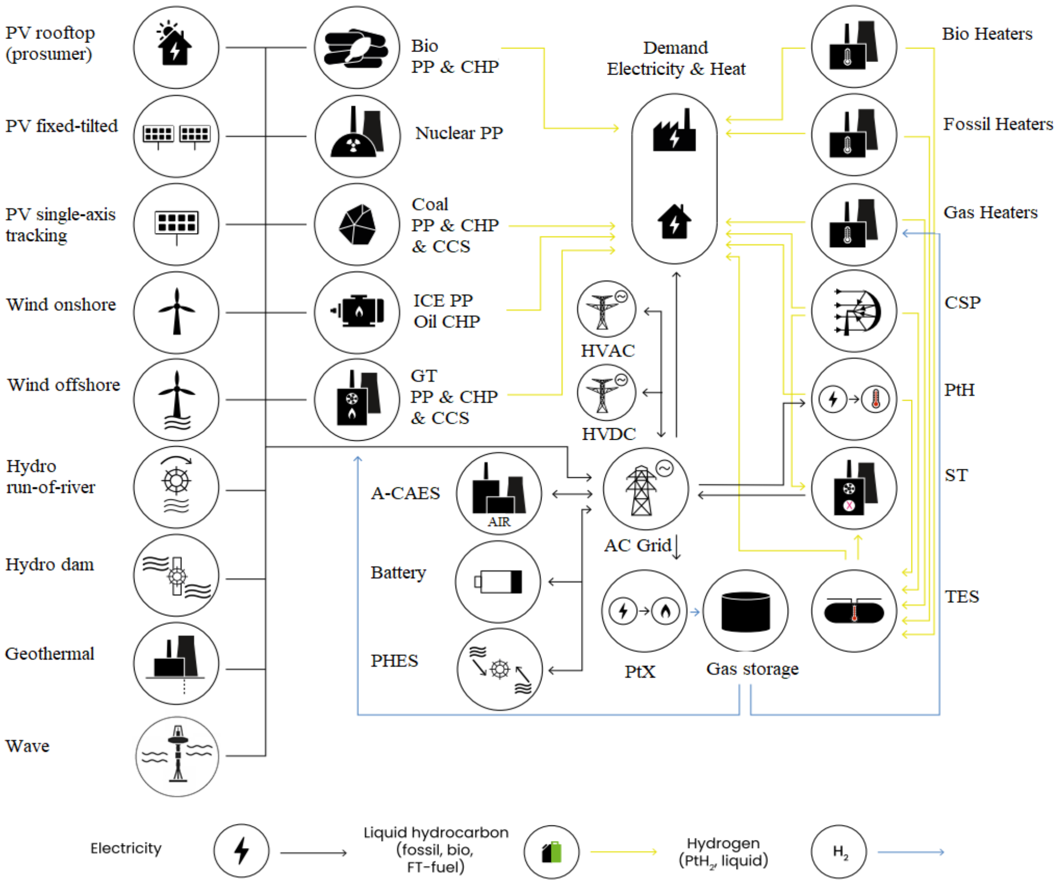

Supplementary Fig. 2. The schematic representation of technologies included in LUT-ESTM. The diagram is in an analogy of [4] and is a modified version of [1]. At the bottom, the commodities are shown. Abbreviations: PP, power plant, CHP, combined heat and power plant, ICE, internal combustion engine, GT, gas turbines, CCS, carbon capture and storage, ST, steam turbines, A-CAES, adiabatic compressed air storage, PHES, pumped hydro energy storage, PtX, power-to-X, CSP, concentrated solar thermal power, PtH, power-to-heat, TES, thermal energy storage, AC, alternating current, HVAC, high voltage alternating current, HVDC, high voltage direct current, FT, Fischer-Tropsch.

1.2 Technologies

The five main categories are identified in LUT-ESTM: Electricity generation (renewable energy, fossil-fuelled and nuclear-based technologies), heat generation (renewable energy and fossil-fuelled technologies), energy storage, energy sector bridging, and electricity transmission. Since the focal point of this paper is the power sector, electricity generation technologies are considered. Due to the nature of the system processes, the energy storage and related auxiliary units (especially, power-to-X technologies and gas storage) are also covered in the analysis.

Electricity generation technologies consist of renewable energy, fossil-fuelled, and nuclear-based technologies. Renewable energy technologies are identified as photovoltaic power plants (optimally fixed-tilted, single-axis tracking, and rooftop), wind power plants (onshore and offshore), hydropower plants (reservoir-dam and run-of-river), wave energy converters, geothermal power plants, fuel cell CHP, methane CHP, waste-to-energy (WtE) power plants CHP, and bioenergy (solid biomass, biogas, and their integration with CHP). Fossil fuelled power plants are classified as coal power plants, ICE, multifuel ICE, open-cycle gas turbines (OCGT), combined-cycle gas turbines (CCGT), oil CHP, and their integration with CHP and CCS units. In addition, concentrated solar thermal power parabolic fields and related components are included in the analysis since their electricity output feeds to the AC grid.

Energy storage units are categorised according to their storage period: Short-term storage (lithium-ion batteries and PHES), medium-term storage (A-CAES, TES district heat (DH) and high temperature (HT)), and long-term storage (power-to-synthetic natural gas (PtSNG) technologies). For this analysis, electrolysis, direct air capture (DAC) from the atmosphere, and methanation units are considered as the main parts of PtSNG technologies for the power sector. Methane (CH_4), carbon dioxide (CO_2), and hydrogen (H_2), are stored in gaseous form during the processes.

The list of technologies is given in the excel file provided as supplementary material. Further information related to the model can be found at Bogdanov et al. [4].

Supplementary Note 2

2 Cumulative Energy Demand Database for LUT-EROI

The advanced systemwide energy return on invested model, constructed at LUT university and thus referred to as LUT-EROI for short, requires a large magnitude of input data of various kind as detailed in the main paper. This section summarises how the cumulative energy demand (CED) data of technologies modelled in LUT-ESTM together with the energy required to operate these technologies are compiled.

2.1 Selection of the system model

The life cycle inventory (LCI) system model is used. The LCI model defines both the unit processes of a system and demonstrates the input-output relationships between the processes[7,8]. LCI is often referred to as the foreground of the model. They are specific to the system; however, in the background, every process in the system is defined and adapted. Therefore, using a reliable database, the selection of appropriate methodology for the calculation, and identification of the system boundaries are crucial for defining LCI inventory of a system.

The most comprehensive and widely used, ecoinvent database (Version 3.7.1.) was selected in order to create a CED database for the LUT-EROI model. In the new version of the ecoinvent database, the system model selection was reshaped considering the three main model concepts: subdivision, allocation, and substitution. The allocation model was selected based on the LUT-ESTM architecture and the principle of its operation. The allocation model is a method applied to transform multi-product activities into single-product activities according to the attributional approach. Three subtypes of the allocation model are described under the ecoinvent database. These are allocation, cut-off by classification, allocation, cut-off, EN15804, and allocation at the point of substitution (APOS).

The APOS sub-system model is a system expansion model, and does not permit the allocation for treatment systems[8–10]. The Allocation, cut-off, EN15804 subsystem model has been recently developed considering EN15804 requirements[11]. This model allows us to choose cut-off points between the primary and secondary systems, prioritising the increase of the recycling features of the system[12]. Conversely, in the allocation, cut-off by classification sub-system model, primary materials are allocated to the primary users. Recycling materials and waste products do not carry significant economic value to impact the system[8,9]. LUT-ESTM is a comprehensive model that has a complex structure, which requires enormous data related to recycling of the materials and waste products, which is hard to obtain on a technology level. At the same time, several questions, such as issues of setting proper boundary conditions, need to be answered before considering an analysis of the impact of recycling on EROI values as system changes. Thus, the most appropriate subsystem model for this analysis was founded to be as allocation, cut-off by classification.

2.2 Identification of system boundary

Life cycle assessment (LCA) is a comprehensive method assessing the environmental impacts in terms of magnitude, types, and potentials throughout the entire lifecycle of a product or a system under specific conditions. The impacts are measured in four main categories: materials sourcing, manufacturing of the materials and/or auxiliary substances, utilisation and recovery processes, and waste management, where these are also referred to as life cycle process flows. By their very nature, these streams (flows) are unlimited in terms of manufacturing, processing, and utilisation ways, and as a result require enormous specific data. For this reason, setting boundaries, interpreting the unit processes' flows, and linking each process together are key steps during the analysis[13–16].

ISO standards EN 14040[17] and EN 14044[18] do not impose any strict requirements for drawing system boundaries for a process or a system, opening the way for various interpretations for the same systems. The restrictions and extensions of the system boundary can be configured according to expert knowledge and anticipation. This can lead to the exclusion of processes and/or flows that have a high impact on the analysis[15]. In this context, three concepts of system boundaries for LUT-EROI have been identified: (i) the boundary between the entire model system (including all unit processes and flows) and the associated environment (natural resources), (ii) the boundary between unit processes (cut-off), and (iii) the boundary between considered unit processes under consideration and other auxiliary systems, whereby multi-product activities are unified into single-product activities (allocation)[16]. Centred on these concepts, the system boundary of LUT-EROI (Supplementary Fig. 3.) was designed according to the following criteria:

- Identification of unit processes (power plants) and their complex structure (including necessary ancillary processes, material and energy flows),
- Required raw material extraction up to component processing,
- Transport of the processed fuels,
- Necessary pre-treatment unit processes,
- Operating principle of unit processes,
- Interoperability of the unit processes,
- Maintenance facilities of individual unit processes,
- Integration of unit processes into the grid system,
- Selection of the electricity delivery point (to the grid, and end-users).

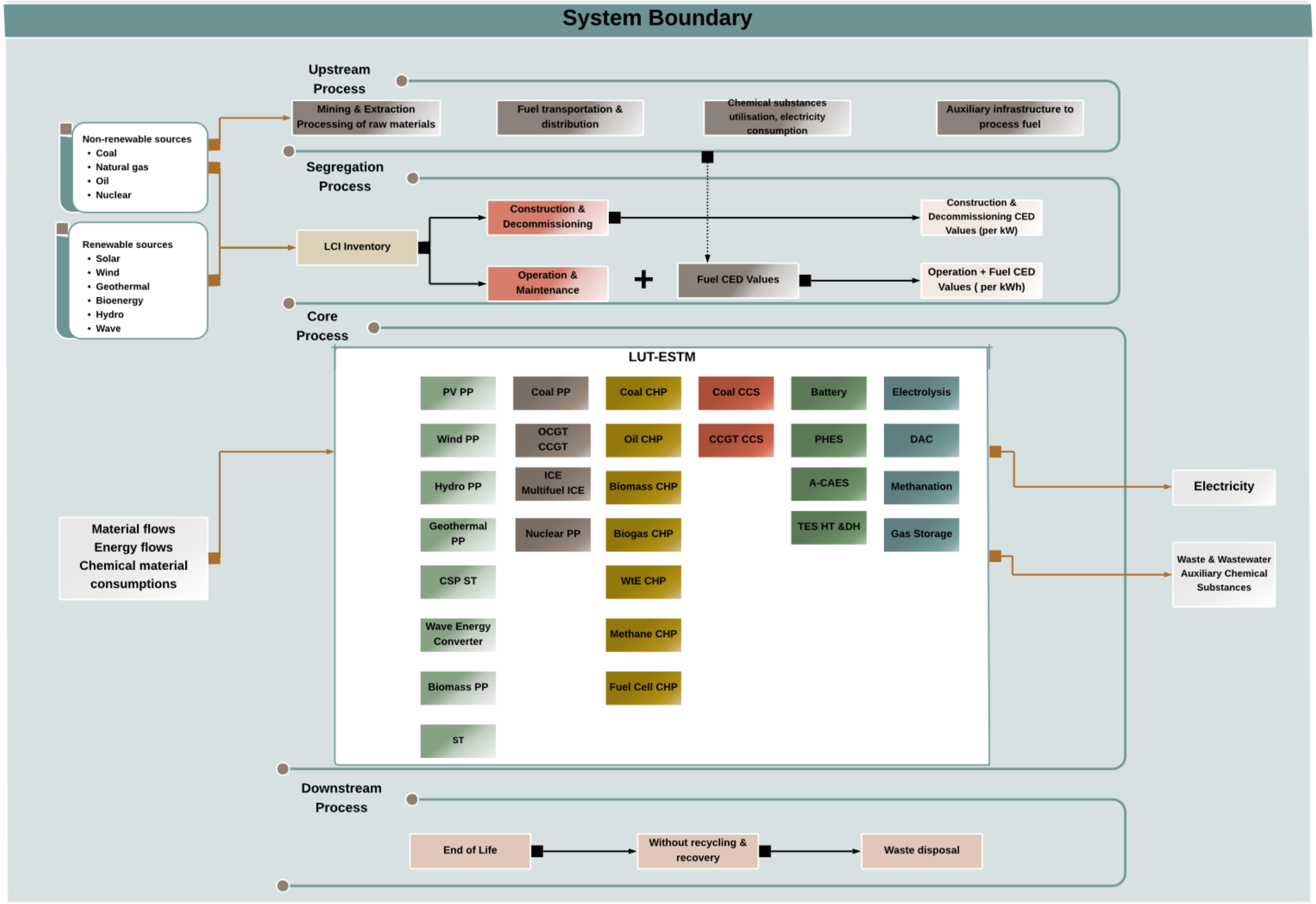

Supplementary Fig. 3. The system boundary for invested energy and systemwide EROI estimation for LUT-ESTM. A-CAES, adiabatic compressed air storage, CCS, carbon capture and storage, CED, cumulative energy demand, CHP, combined heat and power plant, CSP, concentrated solar thermal power, DAC, direct air capture, GT, gas turbines, ICE, internal combustion engine, LCI, life cycle inventory, PHES, pumped hydro energy storage, PP, power plant, ST, steam turbines, TES DH &DH, thermal energy storage high temperature & district heating.

2.3 Cumulative energy demand as an indicator

The cumulative energy demand (CED) refers to the primary energy consumption over the life cycle of a product or a system. It is one of the key impact indicators to estimate the scale and potential impacts of a system from the energy perspective[19]. In LCA, CED focuses exclusively on direct, indirect, and grey energy consumption. Compared to EN 15643-2[20] and EN15978[21], the CED analysis follows a comprehensive and harmonised approach to the life cycle process flows for a system[22,23]. In the ecoinvent database, CED analysis values are disaggregated into 8 sub-categories based on contribution of energy resource type, which is given in units of MJ-equivalents[19].

The CED values of a system have been applied to determine energy return on investment (EROI) analysis; however, the misclassification of energy flows, inconsistency in the energy quality and utilisation efficiency, and the uncertainty of the system boundaries cause to yield various results even for the same type of systems. Therefore, pioneer studies focusing on the relationships of CED and EROI analysis[24–33] have tried to increase the accuracy of the EROI analysis and find a common approach in terms of energy perspective.

2.4 Applying same energy quality by converting all primary energy category to electricity

Traditional primary energy quantification (PEQ) was partly blamed for poor comparability of EROI[24,25,28,29], particularly for renewable energy technology versus fossil fuel. To solve this, researchers recommended using a constant conversion factor[24,25,28,29]. A recent study that explored the PEQ problem has shown that one can achieve a comparable analysis of various technologies and systems by estimating primary energy for all resources at a standard energy quality, for which electricity was selected[31]. Hereby, the primary electricity conversion factors[32–35] are presented in Supplementary Table 1, where $MJ_{pe\text{-}eq}$ units are converted to MJ_{el} according to conversion factors ($MJ_{el} = MJ_{pe\text{-}eq} \cdot$ (conversion factor)) corresponding to each resource types.

Supplementary Table 1. Primary to electricity conversion factors relevant to ecoinvent database[32–35].

	Conversion factor
**Renewable energy resources, biomass**	0.3
**Renewable energy resources, geothermal**	0.1
**Renewable energy resources, solar**	0.9
**Renewable energy resources, potential (in barrage water)**	1.0
**Renewable energy resources, kinetic (in wind)**	0.9
**Non-renewable energy resources, fossil**	0.4
**Non-renewable energy resources, nuclear**	0.3
**Non-renewable energy resources, primary forest**	0.3

2.5 Unit conversion factors

Few materials units were converted by using the following main conversion factors to comply with the ecoinvent database units. These are:

- 1 litre of oil engine is assumed to be 0.885 kilogram (kg)[36].
- 1 litre of glyphosate is assumed to be 0.36 kg[37].
- 1 m^3 of concrete is assumed to be 2.5 metric tonnes[38].
- 1 kWh of electricity is assumed to be 3.6 MJ_{el}.

2.6 Overcoming the challenges of CED value calculations

The conceptual discrepancy of the LCA and CED methodologies, geographical variations, and spatial distribution of resources, major differences of raw material and resource quality, double-counting issues, identification of waste products, and separation of recycling rates are the grand challenges in calculating CED values. These limitations were overcome by using regional level data (GLO, global level; RoW, rest of the world, and RER, European level), categorising the technical data and materials for similar systems, and comparing the literature findings to the estimated CED values. To achieve global scale application, market-level data was preferred to ensure homogeneity for the estimated CED values. If a specific technology is not included in the ecoinvent database, an attempt is first made to create the inventory from technology data sheets for the generation of 1 kilowatt-hour (kW-h) of electricity from selected resources. If this data is not presented in the database, then certain technologies LCA inventories presented by the companies or international projects were used through the publications.

The developed methodology for this study is presented in Supplementary Fig. 4.

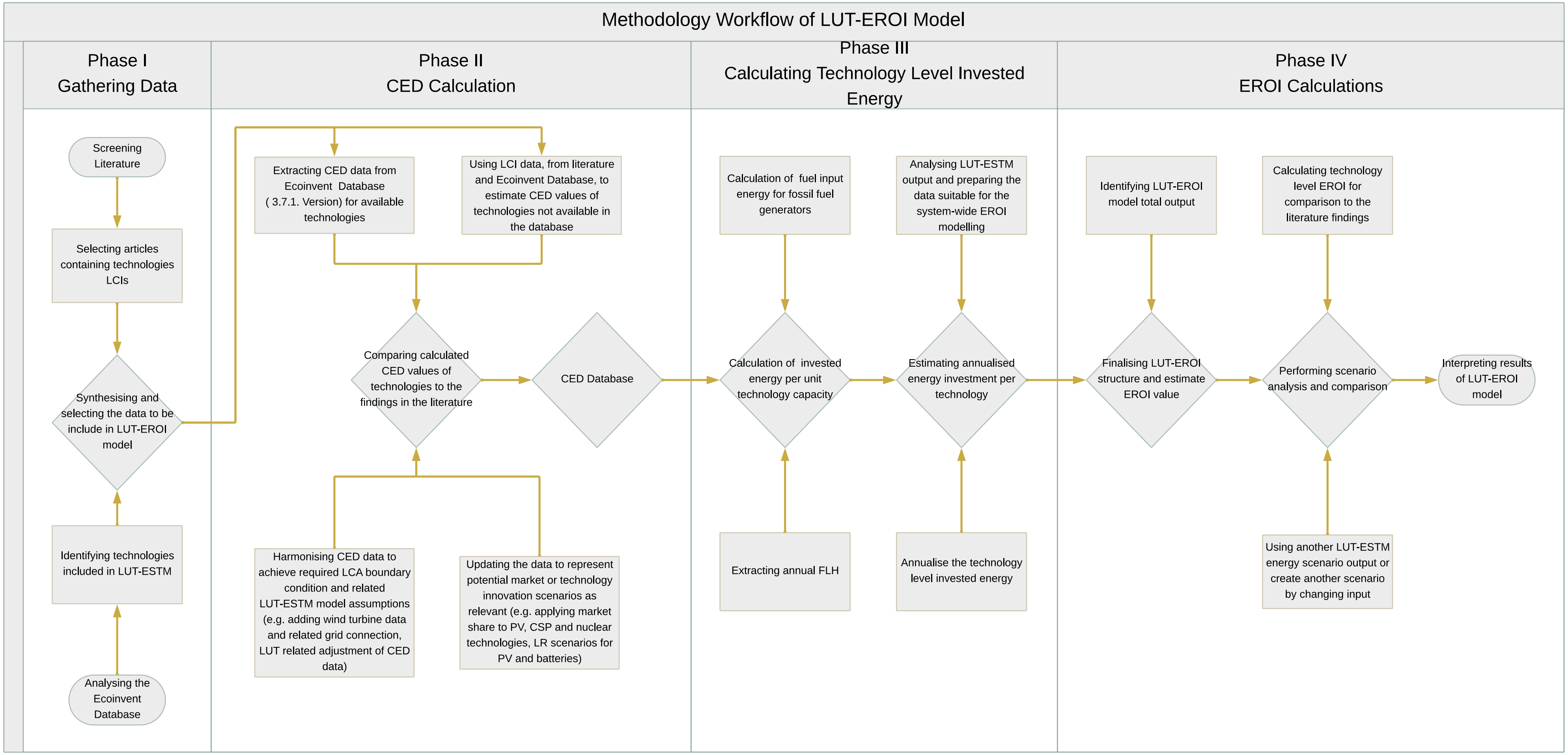

Supplementary Fig. 4. The methodology workflow diagram of the LUT-EROI model. CED, cumulative energy demand, CSP, Concentrated solar thermal power, EROI, energy return on investment, LCI, life cycle inventory, LR, learning rates, PV, photovoltaic.

Supplementary Note 3

3 Cumulative Energy Demand Analysis on Technology Level

3.1 Renewable energy power plants

3.1.1 Photovoltaic (PV) power plants

Optimally fixed-tilted, single-axis tracking, and prosumer (rooftop) are the photovoltaic power plant types covered in LUT-ESTM. In order to find a reference CED for PV power plants, five commercially available PV power plant types are considered in this analysis since the ecoinvent database does not cover all available technology types on the global market. CED values of these system types were extracted from the ecoinvent database and converted as MJ from primary energy to electricity. The balance of system for tracking PV system may require slightly more material than that of fixed tilted PV. However, our literature analysis shows that the CED difference between fixed-tilted PV and tracking system remains roughly about 5%[39,40] as compared to the difference with dual axis, which is approximately 19%[39]. Single-axis tracking is gaining attractiveness in the last few years, which could provide an opportunity to reduce this gap. At the same time, the 5% CED values difference for fixed-tilted PV and single-axis tracking could be observed even for the fixed-tilted PV CED value when different literature is compared, thus this study uses the CED value of fixed-tilted PV for single-axis PV. However, future studies should evaluate the impact if CED gap with proper data if this does not improve. Supplementary Table 2 presents the CED values of the five technologies collected from ecoinvent in units of MJ_{el}. PV CED was improving with time, thus the reference year corresponding to this data were estimated as given in the table after comparing corresponding ecoinvent data of historical technology efficiency and CED values with literature data such as[41,42].

Supplementary Table 2. CED values of PV plants in units of MJ_{el}.

	**Construction & Decommissioning**	**Operation**	**Reference Year**	**Relevant ecoinvent dataset**
	**CED Value (MJ_{el} /kW)**	**CED Value (MJ_{el} /kWh)**		
**Photovoltaic plant, open ground, 570 kWp**	15 747.1	N/A	2012	market for photovoltaic plant, 570 kWp, multi-Si, on open ground (GLO)
**Flat-roof installation, 3 kWp, single-Si, on roof**	17 871.6	N/A	2011	market for photovoltaic flat-roof installation, 3 kWp, single-Si, on roof (GLO)
**Amorphous silicon (a-Si), 3 kWp, panel, mounted on roof**	13 178.5	N/A	2011	market for photovoltaic slanted-roof installation, 3 kWp, a-Si, panel, mounted, on roof (GLO)

**Copper indium selenide (CIGS), 3 kWp, panel mounted on roof**	11 665.5	N/A	2011	market for photovoltaic slanted-roof installation, 3kWp, CIGS, panel, mounted, on roof (GLO)
**Cadmium telluride (CdTe), 3 kWp, laminated, mounted on roof**	10 691.0	N/A	2011	Derived from following datasets: market for photovoltaic slanted-roof installation, 3 kWp, CdTe, laminated, integrated, on roof (GLO) market for photovoltaic slanted-roof installation, 3kWp, a-Si, laminated, integrated, on roof (GLO) market for photovoltaic slanted-roof installation, 3kWp, a-Si, panel, mounted, on roof (GLO)

PV technology module performance continuously increased [42,43], at the same time, the industry has improved its material need for the balance of systems. Such combined enhancement was shown to result in CED values decreases[19,32,44]. Considering the improvement effect, CED values were recalculated using an average energy learning rate (ELR) of 14% until 2030 applying the ELR estimate of, Görig and Breyer[45] and slightly decreased ELR after that as given in Supplementary Table 3. Application of the energy learning curve was performed by using the following Supplementary Equation 1[45].

$$CED_{\text{estimated year}} = CED_{\text{reference year}} \cdot \left(\frac{\text{Cumulative capacity of the estimated year}}{\text{Cumulative capacity of the reference year}}\right)^{\frac{\log(1\text{-ELR})}{\log(2)}} \quad (1)$$

The cumulative capacities from 2009 to 2020 were taken from the REN21 report[46], while future cumulative capacities were projected based on the assumptions of the compound annual growth rate (CAGR) of SolarPower Europe until 2025[47] and extended by the value given in Supplementary Table 3 for reference scenario.

Supplementary Table 3. Reference PV CED values according to years.

	Assumed Capacity	Energy Learning Rates	CAGR	Construction & Decommissioning	Operation
	(GW)	(%)	(%)	CED Value (MJ_{el}/kW)	CED Value (MJ_{el}/kWh)
**2015**	228.0	14%	14%	13 024.9	N/A
**2020**	760.0	14%	14%	10 271.5	
**2025**	1 843.5	14%	17%	8 531.6	
**2030**	3 871.9	14%	16%	7 548.9	
**2035**	7 133.7	13%	13%	6 906.1	
**2040**	11 488.9	13%	10%	6 545.6	
**2045**	17 677.1	12%	9%	6 305.4	
**2050**	23 656.0	12%	6%	6 269.9	

Supplementary Table 4. The global annual production shares of PV module technologies from 2015 to 2050.

PV Plant Technologies	Cadmium telluride (CdTe)	Copper indium selenide (CIGS)	Amorphous silicon (a-Si)	Crystalline silicon (c-Si)	Monocrystalline silicon (mono-Si)
**2015**	4.2%	1.9%	0.6%	68.3%	25.0%
**2020**	4.3%	1.2%	0.1%	26.4%	68.0%
**2025**	4.1%	1.1%	0.1%	14.2%	80.5%
**2030**	3.9%	1.0%	0.1%	5.0%	90.0%
**2035**	3.9%	1.0%	0.1%	4.0%	91.0%
**2040**	4.1%	0.9%	0.0%	3.5%	91.5%
**2045**	4.2%	0.8%	0.0%	3.0%	92.0%
**2050**	4.3%	0.7%	0.0%	2.0%	93.0%

3.1.2 Wind power plants

This study made sure that all power plant level data are based on the full components that includes grid connection, where all these components are estimated based on the cradle to end-of life inventory of the power plant[35]. Thus, wind power plant system estimation was made by combining two ecoinvent data sets, namely wind turbine data set and the corresponding network connection dataset, because ecoinvent market data for wind turbine does not include the grid connection (Supplementary Table 5).

Supplementary Table 5. CED values of wind power plants in units of MJ_{el}.

	Construction & Decommissioning	Operation	Relevant ecoinvent dataset name
	CED Value (MJ_{el} /kW)	CED Value (MJ_{el} /kWh)	
**Wind power plant, 4.5 Megawatt (MW), onshore**	5 404.0	1.49E-03	market for wind turbine, 4.5MW, onshore (GLO)<br>wind turbine network connection construction, 4.5MW, onshore (GLO)<br>market for lubricating oil (RER)<br>market for waste mineral oil (RoW)<br>market for transport, freight, lorry 7.5-16 metric ton, EURO6 (RoW)
**Wind power plant, 2 MW, offshore**	4 423.7	1.56E-03	market for wind power plant, 2MW, offshore, fixed parts (GLO)<br>market for wind power plant, 2MW, offshore, moving parts (GLO)<br>wind turbine network connection construction, 2MW, onshore (RoW)<br>market for lubricating oil (RER)<br>market for waste mineral oil (RoW)

3.1.3 Hydropower plants

Hydropower plants CED values were analysed at the global and country-level, and the data bases for most regions are based on some extrapolation of the inventory data gathered from Switzerland or Canada. This approach appears to be common for other technologies basically because power plant technologies are mostly similar. However, the database selection is mostly impacted by the availability of suitable information to adapt the data LUT-EROI model requirement, for example the possibility of estimating CED value per kW capacity of technologies. The data in Supplementary Table 6 shows the factors determined for hydropower based on this method.

Supplementary Table 6. CED values of hydropower plants in units of MJ_{el}.

	Construction & Decommissioning	Operation	Relevant ecoinvent dataset name
	CED Value (MJ_{el} /kW)	CED Value (MJ_{el} /kWh)	
**Hydropower plant, reservoir, 9130 MW**	6 899.0	2.05E-04	market for hydropower plant, reservoir (GLO)<br>market for lubricating oil (RER)<br>market for waste mineral oil (RoW)
**Hydropower plant, run-of-river, 13,787 MW**	11 212.0	2.05E-04	hydropower plant construction, run-of-river (CA-QC)<br>market for lubricating oil (RER)<br>market for waste mineral oil (RoW)

3.1.4 Geothermal power plant

The selected data includes the energy requirements for the deep well drilling, the consumption of additional chemical substances, and materials for drilling.

Supplementary Table 7. CED values of geothermal power plant in units of MJ_{el}.

	Construction & Decommissioning	Operation	Relevant ecoinvent dataset name
	CED Value (MJ_{el} /kW)	CED Value (MJ_{el} /kWh)	
**Geothermal power plant, 5.5 $MW_{el.}$**	133 766.8	9.13E-05	market for geothermal power plant, 5.5MW_{el}. (GLO)<br>market for benzene (GLO)

3.1.5 Wave energy converter

There are only a few publications examining the life cycle assessment of wave energy converters (WECs)[48–51]. In this analysis, the CorPower Ocean AB point absorber WEC was selected based on its availability in the global market and maturity level in terms of TRL. The life cycle inventory of the WEC was

taken from Pennock et al.[52] and material consumption values were adapted considering the total weight of WEC, which is 45 metric tonnes per array[53].

Supplementary Table 8. CED values of wave energy converter in units of MJ_{el}.

	Construction & Decommissioning	Operation	Relevant ecoinvent dataset name
	**CED Value (MJ_{el} /kW)**	**CED Value (MJ_{el} /kWh)**	
**Wave energy converter, 10 MW**	941.6	2.87E-04	market for steel, chromium steel 18/8 (GLO)<br>market for glass fibre reinforced plastic, polyamide, injection moulded (GLO)<br>market for copper, cathode (GLO)<br>market for aluminium, cast alloy (GLO)<br>market for polyethylene, high density, granulate (GLO)<br>market for transport, freight, lorry 16-32 metric ton, EURO6 (RoW)<br>market for transport, freight, sea, ferry (GLO)<br>market for heavy fuel oil (RoW)

3.1.6 Bioenergy related power plants

Estimating the biomass and biogas input characteristics (calorific value, quantity etc.) is challenging due to their production in multiple sectors (agriculture, forestry, waste, marine, bio-based industries), their geographical and spatial distribution, and their mobilisation[54]. The total electricity generation from these power plants is very sensitive to the content and condition of the input mixture, the amount and the calorific value of the input because the mixing ratio and properties of the input are not stable[55–57]. Under these circumstances, the calculation of the CED values for the power plants can only be carried out using samples. The inventory data for these power plants was compiled from the selected literatures considering that it presents reliable and detailed data content, the market developments of the specific technology, and the degree of the pilot projects' implementation. The pilot project data taken from companies were adopted to approximate the real situation.

A UK case study[58,59] was selected to calculate CED values of the biomass power plant and biomass co-generation and heat power (CHP) plant. The reasons for choosing this literature are: (i) implementation of the gasification technology, (ii) presentation of detailed data on material consumption and types, (iii) handling of different types of inputs (willow and miscanthus) and providing specific data for each input types, (iv) providing an inventory of previous steps (soil pre-treatment, harvesting, collection, etc.) regarding inputs, (v) including a separate net energy analysis for these power plants, and (vi) presenting data taken from a real case.

A CED value for the wood combustion unit was derived[58,59] considering the system boundary of the biomass power plant. The inventory related to the preparation of willow, (soil pre-treatment, chemical types and their consumption amounts, diesel and oil consumption for agricultural machines etc.) was assumed to be

the same, hence, the CED operation value is estimated. The required cultivation area for willow was given as 62.5 hectare[58], with willows cultivated seven times over a period of 23 years. Therefore, the diesel and oil consumptions for agricultural machines, and the chemical consumption amounts for the pre-treatment operations were determined considering the cultivation period. The lifetime of the gas engine is 20 years and the lifetime oil consumption of the gas engine was calculated as 2,198.3 kg in relation to the assumed operation[59]. The detailed datasets are presented in the Supplementary Note 5 section, where the used material types are provided for each unit. The amounts related to material usage and technical details are taken from the supplementary materials of the studies[58,59].

Biogas CHP plant CED values were estimated using the heat and power co-generation, biogas, gas engine technology database included in the ecoinvent database[35]. As stated in the technology database pdf file, inputs from the technosphere are limited to the gas engine unit for electricity generation and lubricating oil consumption for this unit[35]. To be consistent with the structure of the LUT-ESTM biogas CHP plant and its system boundary conditions, biogas power plant units were included and adapted to 160 kW capacity (Supplementary Table 9). Note that the lubricating oil and waste mineral oil amounts are converted according to the use of one unit gas engine.

The anaerobic digestion technology has become a more significant source of energy and heat generation in various countries around the world. The world's biogas electricity generation is contributed by Europe 75%, and is followed by North America 17%, Asia 5%, Eurasia 2%, South America 1.1%, and Africa 0.1%. The prominence of anaerobic digestion plants among other biogas related technologies is due to its ability utilise a wide range of organic matter (from food waste, municipal and industrial sewage, agricultural materials and livestock manures) to produce energy and heat, and its ease to scale up based on local needs. However, daily collected waste and its composition are crucial parameters when dimensioning the anaerobic digestion plant[60]. For this reason, the global average data of anaerobic digestion plants for biodegradable waste was preferred in the ecoinvent database.

The ecoinvent database[35] specifies a maximum annual waste utilisation capacity of 10 kiloton (kt) for anaerobic digestion plants. 50% of the electricity consumption[61] was assumed to be used for operational purposes, while the other part was used for the construction of the whole structure. The collection and transportation of the waste and the required diesel consumption for the transportation were recalculated using the input data from the WtE CHP[62], which are 2,135.4 metric tonnes*kilometre (km)/year, and 970.6 kg/year, respectively.

Supplementary Table 9. CED values of bioenergy related power plants in units of MJ_{el}.

	Construction & Decommissioning	Operation	Relevant ecoinvent dataset name
	**CED Value (MJ_{el} /kW)**	**CED Value (MJ_{el}/kWh)**	
**Biomass power plant, 230 kW**	862.7	2.67E-01	Wood combustion unit (Supplementary Note 5, Supplementary Table 36)
**Biogas power plant, 230 MW**	1 554.4	2.77E-01	anaerobic digestion plant construction, for biowaste (GLO)<br>market group for electricity, medium voltage (GLO)<br>market for municipal waste collection service by 21 metric ton lorry<br>market for diesel (RoW)<br>biogas upgrade unit
**Biogas CHP, 160 kW**	2 133.4	2.49E-01	market for heat and power co-generation unit, 160kW electrical, components for electricity only (GLO)<br>market for lubricating oil (RER)<br>market for waste mineral oil (RoW)<br>Biogas power plant (Supplementary Table 9)

3.1.7 Waste-to-energy CHP plant

The municipal waste incineration facility is determined as the main unit for the WtE CHP plant. The required chemicals and electricity consumption values[63] were adjusted considering the annual waste processing capacity of the power plant. A 21 metric tonnes lorry was selected for the transportation and collection of the waste[62]. It is assumed that the lorry can carry 10.5 tonnes per waste collection[62] whereas vehicle-kilometre is 5.38 metric tonne-kilometre. The frequency of collection was estimated considering the amount of waste collected during the lifetime of the facility while it is operated continuously 365 days per year.

Similar to bioenergy related power plants, the waste composition, quantity, and lower heating value are important to determine the capacity of this plant[55,56]. In the ecoinvent database, the waste incineration facility is provided as a unit, which indicates the annual waste processing capacity instead of the net electrical installed capacity. Therefore, the gross electrical installed capacity of the WtE CHP plant was calculated using the following Supplementary Equation 2[64].

$$EPP_{msw} = 277.8 \cdot LHV_{msw} \cdot \left(\frac{\omega_{msw}}{24}\right) \cdot \eta \qquad (2)$$

where EPP_{msw} is the estimation of the electric power potential of the municipal solid waste (as kW), the net lower heating value of MSW (MJ/kg) is LHV_{msw} , ω_{msw} is the weight of municipal solid waste, η is the conversion efficiency of the power plant (taken as 30%)[64]. The installed gross electricity capacity was

calculated using the lower heating value (LHV) (17.65 MJ/kg) and the annual waste incineration capacity (100,000 tonnes/year)[35]. The installed gross electrical capacity was found as 16.8 MW. Excluding the self-electricity consumption of the power plant, the net electrical installed capacity (GP_{MW}) of the WtE CHP plant was determined by applying Supplementary Equation 3[64].

$$GP_{MW} = EPP_{msw} \cdot \eta_G \cdot \eta_P \cdot \frac{1}{1000} \quad (3)$$

In Supplementary Equation 3, η_G and η_P refer to generator efficiency and transmission efficiency, respectively. The generator efficiency was assumed to be 90% whereas steam turbine efficiency was taken as 75%[64]. Hence, the installed net electricity capacity of the WtE CHP plant was found as 11.3 MW. 50% of total CED values are assumed to be used for electricity generation and heat production, while the other 50% are assumed to be related to the primary purpose of waste incineration, but not energy supply. Based on this percentage, 70% of the electricity and heat supply CED value is allocated for only electricity generation.

Supplementary Table 10. CED values of WtE CHP in units of MJ_{el}.

	**Construction & Decommissioning**	**Operation**	**Relevant ecoinvent dataset name**
	**CED Value (MJ_{el} /kW)**	**CED Value (MJ_{el} /kWh)**	
**Waste-to-energy power plant CHP, 7.5 MW (including the waste treatment, electricity generation and heat production)**	9 671.3	2.41E-01	market for municipal waste incineration facility (GLO)<br>market for municipal waste collection service by 21 metric ton lorry (GLO)<br>market for diesel (RoW)<br>market for quicklime, milled, loose (RoW)<br>market for sodium hydroxide, without water, in 50% solution state (GLO)<br>market for activated carbon, granular (GLO)<br>market for ammonia, anhydrous, liquid (RoW)<br>market for neutralising agent, sodium hydroxide equivalent (GLO)<br>market group for electricity, medium voltage (GLO)
**Waste-to-energy power plant CHP, 7.5 MW (including only electricity generation and heat production)**	3 205.3	7.97E-02	
**Waste-to-energy power plant CHP, 7.5 MW (including only electricity generation)**	2 243.7	5.58E-02	

3.1.8 Methane CHP plant

There is no specific data for methane CHP in the ecoinvent database. Therefore, considering the similarity of the systems and operating principles, it is assumed that the CED value of methane CHP is the same as that of oil CHP provided in the ecoinvent database[35].

Supplementary Table 11. CED values of methane CHP plant in units of MJ_{el}.

	Construction & Decommissioning	Operation	Relevant ecoinvent dataset name
	CED Value (MJ_{el} /kW)	CED Value (MJ_{el} /kWh)	
**Methane CHP, 500 MW**	1 601.7	1.33E-02	market for bilge oil (RoW)<br>market for municipal solid waste (RoW)<br>market for oil power plant, 500MW (GLO)<br>market for waste mineral oil (RoW)<br>market for ammonia, anhydrous, liquid (RoW)<br>market for fly ash and scrubber sludge (RoW)<br>market for lignite ash (RoW)<br>market for limestone, crushed, washed (RoW)<br>market for residue from cooling tower (RoW)<br>market for water, completely softened (RoW)<br>market for water, decarbonised (RoW)

3.1.9 Fuel cell CHP plant

The CED values of this technology were estimated based on low pressure polymer electrolyte membrane (PEM) fuel cell $2kW_e$, future in the ecoinvent dataset[35]. Although the technical data is given in kWh functional units focusing electricity output from a $2kW_e$ PEM fuel cell, the data was adjusted to conform to the functional units of LUT-ESTM.

Supplementary Table 12. CED values of fuel cell CHP plant in units of MJ_{el}.

	Construction & Decommissioning	Operation	Relevant ecoinvent dataset name
	CED Value (MJ_{el} /kW)	CED Value (MJ_{el} /kWh)	
**Fuel cell CHP, 2 kW_e**	13 867.3	4.91E-03	market for fuel cell, polymer electrolyte membrane, 2kW electrical, future (GLO)<br>market for maintenance, polymer electrolyte membrane fuel cell, 2kW electrical (GLO)<br>market for storage, 650 l mini-CHP plant (GLO)

3.1.10 Concentrated solar thermal power (CSP) plant

The reference CED values of CSP plants do not include a thermal storage system. Therefore, the values extracted from the ecoinvent database[35] were altered considering the CSP system boundary condition of LUT-ESTM.

Supplementary Table 13. CED values of CSP ST in units of MJ_{el}.

	Construction & Decommissioning	Operation	Relevant ecoinvent dataset name
	CED Value (MJ_{el} /kW)	CED Value (MJ_{el} /kWh)	
**Concentrated solar thermal power plant, 50 MW (parabolic trough)**	12 906.9	N/A	market for concentrated solar power plant, solar thermal parabolic trough, 50 MW (GLO)<br>market for thermal storage system, solar thermal parabolic trough, 50 MW (GLO)
**Concentrated solar thermal power plant, 20 MW (solar tower)**	13 451.1	N/A	market for concentrated solar power plant, solar tower, 20 MW

The historical and projected cumulative installed CSP capacities for the periods 2010-2020 and 2030-2050 were taken from the REN21 report[46] and the World Energy Outlook report[65], respectively. The missing capacities for the years 2025, 2035, and 2045 were filled by trend analysis of the second-order polynomial function. The CAAGR values were found to proximate IEA's estimations, which are 22.0% for the 2020-2030 period, and 15.0% for the 2020 to 2050 period[66]. In this case, the estimated CAAGR values were 21.6% for the 2020-2030 period, and 15.5% for the 2020-2050 period which is quite close to the IEA's estimates.

The data extracted from the World Bank report[67] and National Renewable Energy Laboratory (NREL) website[68] was classified according to the location, technology type, storage hours, current status, and the expected operation years to identify the future prominent technology. The shares of CSP plant technologies based on cumulative capacity from 2015 to 2050 is given in Supplementary Table 14. The reference CED of the CSP plant (Supplementary Table 15) was estimated based on shares of technology types, CAGR values and future capacity estimations.

Supplementary Table 14. The cumulative capacity shares of CSP plant technologies from 2015 to 2050.

CSP Plant Technologies	Parabolic Trough (%)	Solar Tower (%)	Others (%)
**2015**	85.1%	11.6%	3.3%
**2020**	73.9%	18.5%	7.6%
**2025**	70.3%	20.8%	8.9%
**2030**	55.0%	39.4%	5.6%
**2035**	55.0%	39.4%	5.6%
**2040**	55.0%	39.4%	5.6%
**2045**	55.0%	39.4%	5.6%
**2050**	55.0%	39.4%	5.6%

Supplementary Table 15. Adjusted CSP CED values according to years in units of MJ_{el}.

	Cumulative Installed Capacity	Average CAGR	Construction & Decommissioning	Operation
	(GW)	%	CED Value (MJ_{el} /kW)	CED Value (MJ_{el} /kWh)
**2015**	4.6	−	12 988.3	N/A
**2020**	6.2	1.6 %	13 048.9	
**2025**	23.6	17.1%	13 068.3	
**2030**	46.0	14.0%	13 152.1	
**2035**	138.3	15.5%	13 152.1	
**2040**	232.0	8.9%	13 152.1	
**2045**	327.3	6.2%	13 152.1	
**2050**	424.0	4.8%	13 152.1	

3.1.11 Steam turbine (ST) component

The power block for the 50 MW CSP parabolic trough plant was obtained from the ecoinvent database[35]. The CED value of operation was assumed to be zero due to the lack of data.

Supplementary Table 16. CED values of ST in units of MJ_{el}.

	Construction & Decommissioning	Operation	Relevant ecoinvent dataset name
	CED Value (MJ_{el} /kW)	CED Value (MJ_{el} /kWh)	
**Power block, solar thermal parabolic trough, 50 MW**	3 847.6	N/A	market for power block, solar thermal parabolic trough, 50 MW (GLO)

3.2 Fossil-fuelled power plant

3.2.1 Coal based power plants

In the ecoinvent database, hard coal power plant CED values are represented as market data provided by the global average. CED value belongs to construction and decommissioning was taken from the market for hard coal power plant technology database. Based on the annual production volumes of 100 MW and 500 MW hard coal power plants, the average capacity of the hard coal power plant was determined.

The operational CED value of hard coal power plant was derived from the hard coal CHP dataset, which provides the corresponding estimates in units of kWh by allocating the values to the electricity output of hard coal CHP. The substances consumption amounts were adjusted according to the hard coal power plant and its installed capacity.

Hard coal CHP plant CED values (Supplementary Table 17) were calculated by adapting the heat and power co-generation, hard coal technology database. The technology data is provided for the generation of 1 kWh of electricity, thereof, all amounts of materials and substances were adapted depending on the unit amount of the plant.

The CCS unit LCI data consists of CO_2 capture infrastructure, CO_2 compressor infrastructure, CO_2 pipeline infrastructure, CO_2 injection facility, monoethanolamine (MEA) chemical facility and MEA production. With exception of MEA chemical plant infrastructure[69], the LCI data for CCS infrastructure components were obtained from Koornneef et al. study[70]. The CED values for each sub-infrastructure facility were adjusted according to the installed capacity of the hard coal power plant (455 MW) and its LUT-ESTM lifetime value.

Supplementary Table 17. CED values of coal-based power plants in units of MJ_{el}.

	Construction & Decommissioning	Operation	Dataset
	**CED Value (MJ_{el} /kW)**	**CED Value (MJ_{el} /kWh)**	
**Hard coal power plant, 389.8 MW**	1 399.0	1.62E-02	market for hard coal power plant (GLO)<br>market for hard coal ash (RoW)<br>market for residue from cooling tower (RoW)<br>market for water, completely softened (RoW)<br>market for water, decarbonised (RoW)
**Hard coal power plant with CHP unit, 389.8 MW**	2 861.6	1.62E-02	market for hard coal power plant (GLO)<br>market for hard coal ash (RoW)<br>market for NOx retained, by selective catalytic reduction (GLO)<br>market for SOx retained, in hard coal flue gas desulphurisation (RoW)<br>market for residue from cooling tower (RoW)<br>market for water, completely softened (RoW)<br>market for water, decarbonised (RoW)
**Hard coal power plant with CCS unit, 455 MW**	2 936.3	1.78E-01	market for hard coal power plant (GLO)<br>CCS unit (Supplementary Note 5, Supplementary Table 37)

3.2.2 Gas based power plants

OCGT and CCGT CED values were based on the corresponding ecoinvent market dataset[35]. The extracted values do not cover the operational phases of the power plants, which was estimated by using electricity production, natural gas, conventional power plant and electricity production, natural gas, combined cycle power plant datasets per units of kWh electricity produced from the use of natural gas[35]. The additional substance amounts were recalculated according to gas turbine types and their installed capacities.

To estimate the CCS components for the CCGT plant. The same procedure as for hard coal CCS plant was followed. The amount of chemical substances' consumptions and infrastructure values were adjusted to the installed capacity of the CCGT plant (455 MW) and its LUT-ESTM lifetime value.

Supplementary Table 18. CED values of gas-based power plants in units of MJ_{el}.

	**Construction & Decommissioning**	**Operation**	**Dataset**
	**CED Value (MJ_{el} /kW)**	**CED Value (MJ_{el} /kWh)**	
**OCGT, 100 MW**	326.4	1.68E-04	market for gas power plant, 100MW electrical (GLO)<br>market for residue from cooling tower (RoW)<br>market for water, completely softened (RoW)<br>market for water, decarbonised (RoW)
**CCGT, 400 MW**	1010.9	1.21E-03	market for gas power plant, combined cycle, 400MW electrical (GLO)<br>market for residue from cooling tower (RoW)<br>market for water, completely softened (RoW)<br>market for water, decarbonised (RoW)
**CCGT CCS plant, 455 MW**	2 458.1	1.36E-01	market for gas power plant, combined cycle, 400MW electrical<br>CCS unit (Supplementary Note 5, Supplementary Table 37)

3.2.3 Internal combustion engine (ICE) power plants

The ICE technology can be integrated into the grid system and generate electricity using the derived fossil fuels, but there is not a suitable dataset in the ecoinvent database. Due to the absence of the completed ICE components (diesel engine, alternator, cooling system, exhaust system, air filter, control panel, fuel tank and circuit breaker) in ecoinvent, the LCI data from Cummins Energy Co., Ltd was retrieved from Jiyang[71] and used to estimate CED values.

Supplementary Table 19. CED values of ICE and multifuel ICE in units of MJ_{el}.

	**Construction & Decommissioning**	**Operation**	**Relevant ecoinvent dataset name**
	**CED Value (MJ_{el} /kW)**	**CED Value (MJ_{el} /kWh)**	
**ICE & Multifuel ICE, 823 kW**	401.6	1.68E-04	reinforcing steel production (RoW)<br>market for cast iron (GLO)<br>market for aluminium, primary, ingot (RoW)<br>market for copper, cathode (GLO)<br>market for polyethylene, low density, granulate (GLO)

			market for transport, freight, lorry 16-32 metric ton, EURO5 (RoW)<br>market for freight, sea, container ship (GLO)

3.2.4 Oil CHP plant

Heat and power co-generation of oil power plant data in ecoinvent was used to determine CED values for the oil CHP plant. The materials consumed in the construction, decommissioning and operational phases were segregated to find operation CED value. Parallel to similar technology data processes, designed to generate 1 kWh of electricity from resources, the consumption quantities of the materials and chemical substances were converted to be compatible with a 500 MW oil CHP plant.

Supplementary Table 20. CED values of oil CHP plant in units of MJ_{el}.

	**Construction & Decommissioning**	**Operation**	**Relevant ecoinvent dataset name**
	**CED Value (MJ_{el} /kW)**	**CED Value (MJ_{el} /kWh)**	
**Oil CHP, 500 MW**	1 601.7	1.33E-02	market for bilge oil (RoW)<br>market for municipal solid waste (RoW)<br>market for oil power plant, 500MW (GLO)<br>market for waste mineral oil (RoW)<br>market for ammonia, anhydrous, liquid (RoW)<br>market for fly ash and scrubber sludge (RoW)<br>market for lignite ash (RoW)<br>market for limestone, crushed, washed (RoW)<br>market for residue from cooling tower (RoW)<br>market for water, completely softened (RoW)<br>market for water, decarbonised (RoW)

3.3 Nuclear power plants

The CED values of pressurised water reactor (PWR), boiling water reactor (BWR), small modular reactor (SMR), advanced heavy-water reactor (AHWR), and gas turbine modular reactor (GT-MHR) were collected from various sources. PWR and BWR nuclear power plants CED values were obtained from the ecoinvent database[35], which only covers construction and decommissioning phases. For the SMR type of nuclear power plant, Sizewell B NPP of Westinghouse type, located at Suffolk in the UK, was selected because of its generation II reactor. The selected literature provides detailed LCI data for SMR nuclear power plant[72]. On the other hand, the inventory data of AHWR and GT-MHR nuclear power plants were taken from Ashley et al.[73], however the installed capacities and technical parameters (such as lifetime, electricity generation amount, internal electricity consumption) were derived from another study of Ashley et al. [74].

The data on nuclear power plants in operation and planned were obtained from the World Nuclear Association[75,76], and the detailed data for future capacities and nuclear power plant numbers were extracted

from the International Atomic Energy Agency[77,78]. The reference CED values were found by taking cumulative capacity shares according to nuclear types and multiplying with their specific CED values. It is important to note that some technologies, such as fast neutron reactor, are not included due to lack of data and lack of available commercial technology, but could be represented well with average CED values of AHWR and GT-MHR nuclear power plants.

Supplementary Table 21. CED values of nuclear power plants in units of MJ_{el}.

	**Construction & Decommissioning**	**Operation**	**Relevant ecoinvent dataset name**
	**CED Value (MJ_{el} /kW)**	**CED Value (MJ_{el} /kWh)**	
**PWR, 1000 MW**	4 131.2	9.14E-03	market for nuclear power plant, pressure water reactor 1000MW
**BWR, 1000 MW**	4 309.3	N/A	market for nuclear power plant, boiling water reactor 1000MW
**SMR, 225 MW**	1 800.5	N/A	SMR nuclear power plant (Supplementary Note 5, Supplementary Table 38)
**AHWR, 300 MW**	12 413.5	N/A	AHWR nuclear power plant (Supplementary Note 5, Supplementary Table 38)
**GT-MHR, 286 MW**	4 016.7	N/A	GT-MHR nuclear power plant (Supplementary Note 5, Supplementary Table 38)

Supplementary Table 22. The cumulative capacity shares of nuclear power plant technologies from 2015 to 2050.

**Nuclear Power Plant Technologies**	**PWR**	**BWR**	**FBR**	**SMR**	**Others**
**2015 - 2020**	79.6%	16.0%	0.4%	0.0%	4.1%
**2021- 2030**	81.0%	14.0%	0.7%	0.7%	3.6%
**2031 -2050**	80.4%	13.6%	1.6%	1.2%	3.1%

Supplementary Table 23. Adjusted nuclear power plant CED values according to years in units of MJ_{el}.

	**Cumulative Installed Capacity**	**Construction & Decommissioning**	**Operation**
	**(GW)**	**CED Value (MJ_{el} /kW)**	**CED Value (MJ_{el} /kWh)**
**2015 - 2020**	394.2	4 705.7	9.14E-03
**2021 - 2030**	449.14	4 665.3	
**2031 - 2050**	521.37	4 707.3	

The operation CED value (Supplementary Note 5, Supplementary Table 39) for PWR nuclear power plant was calculated based on electricity production, nuclear, pressure water reactor technology data in the ecoinvent database[35]. It is important to recall that the calculations made on the basis of this type of technology data were

performed without including energy content of the main fuel elements of the systems such as coal, natural gas, diesel, fuel oil, and biomethane. In this case, the energy content of consumed uranium (as main input) was not included in the operation CED value.

PWR nuclear power plant capacity share is expected to reach 80.4% in 2050[77,78]. For this reason, the operational CED value for PWR nuclear power plants was taken as an average value for all types of power plants. Further details are provided in Supplementary Note 5.

3.4 Short-term and medium-term storage systems

3.4.1 Short-term storage systems

Battery technology is continually improving both in performance and cost competitiveness driven by the increase in demand for grid integration of RE and large-scale applications in electric vehicles. Among the battery technologies, lithium-ion batteries (LIB) have taken the lead due it its high specific energy, long cycle life, and high energy efficiency[79,80]. Due to its fast cost decline and high efficiency, large quantities of LIB are built in LUT-ESTM results. The inventory data for various LIB was given in Westlake[81] and Pellow et al.[82].

The utility-scale battery storage system is composed of seven parts: (i) container housing, (ii) battery components, (iii) the power conversion system (PCS), (iv) systems controls and communications, (v) HVAC thermal management system, (vi) fire suppression system, and (vii) additional equipment (main switch, cables, breakers, fuses, other electronic parts). Their material types, weights, and compositions are decided based on Westlake[81], and the system is adjusted for LUT-ESTM. For battery components, the LIB dataset is taken from the ecoinvent database that includes modules, racks, and battery management system (BMS). The weights of the components are adjusted considering the difference in battery cell numbers per module. At the same time, our comparison reveals that the BMS related inventory data in ecoinvent database appears to be a simple component[35,83] as the mass comparison was negligible as compared to the inventory data presented in Westlake[81]. Thus, we used the data of Westlake[81] to represent utility-scale BMS. However, The PCS in Westlake[81] presents the combination of the various components, such as inside cooling system and chemical materials for it, because of a specific configuration according to authors perspective. However, these components could be shared by the battery system if the PCS is housed as a one system depending on its design. To avoid overestimation of the CED by including components that could be potentially shared, the PCS component is represented by an inverter only based on the estimation of Pellow et al.[82]. The auxiliary materials (cables, communication module, switches, etc.) needed for connection of each unit for the battery are simply gathered under electronics for control units. On the other hand, the weight of the liquid storage tank as part of the fire suppression system was converted by using information in Althaus et al.[69].

Supplementary Table 24. CED values of lithium-ion battery in units of MJ_{el}.

	Construction & Decommissioning	Operation	Relevant ecoinvent dataset name
	CED Value (MJ_{el} /kWh)	CED Value (MJ_{el} /kWh)	
**Li-on Battery, 1 kWh**	1654.0[1]	N/A	market for battery, Li-ion, rechargeable, prismatic (GLO)<br>market for intermodal shipping container, 40-foot, high-cube (GLO)<br>market for printed wiring board, for power supply unit, desktop computer, Pb free (GLO)<br>market for inverter, 500kW (GLO)<br>market for ventilation system, central, 1 x 720 m^3/h, steel ducts, with earth tube heat exchanger (GLO)<br>reinforcing steel production (RoW)<br>market for electronics, for control units (GLO)<br>chlorodifluoromethane production (RoW)<br>liquid storage tank production, chemicals, organics (RoW)<br>market for drawing of pipe, steel (GLO)

[1] The value is referred to as Reference CED.

LIB is serving as a promising energy storage technology, having a high potential to be deployed in grid-level applications for both electricity generation companies and in lower specific capacity on prosumer sites. It supports the operation mechanism (via peak shaving, frequency and load management) and facilitates the configuration of the system with its traceability[79,80,84]. Despite its rapid evolution over the past decade, the effects of utilisation rate of raw material, its ageing mechanism, changes in the battery chemistry on the markets[85], and energy policies cannot be fully envisaged and explained. There is no doubt these factors will lead to changes in the CED values of the future technologies. For the LUT-ESTM model, LIB is a crucial part of the system, which is linked to required renewable energy generation growth. To capture the potential improvements in CED values from 2020 to 2050, Supplementary Equation 1 was used for estimates, taking future projections of capacity and learning rates as the main parameters, and setting the reference year to 2015 (Supplementary Table 25).

Xu et al.[84] worked through the projections of the capacities, prioritising the effects of battery chemistry and the market shares of battery technologies. In this analysis, the capacity projection of the sustainable development scenario[84] were taken to recalculate the CED values. Our analysis shows that the projection is like a median capacity to scenarios presented in Greim et al.[86]. As discussed in the main paper, Hsieh et al.[85] estimates that the learning rate of the active material synthesis is approximately 3.5% for established technologies. Thus, we chose a battery CED learning rate at 5.0%, which slowly reduces to 4.0% by 2050. As a result, the CED value of the LIB was reduced from 1654 MJ_{el} /kWh in 2015 to 1046.1 MJ_{el} /kWh in 2050.

Supplementary Table 25. Adjusted battery CED value according to years in units of MJ_{el}.

	Cumulative Installed Capacity	Base Energy Learning Rates	Construction & Decommissioning	Operation
	(GW)	%	CED Value (MJ_{el} /kWh)	CED Value (MJ_{el} /kWh)
**2015**	60.9	5.0 %	1 654.0	N/A
**2020**	986.7	5.0 %	1 345.9	
**2025**	4 819.6	4.5 %	1 237.2	
**2030**	13 191.2	4.5 %	1 157.1	
**2035**	28 238.4	4.0 %	1 152.2	
**2040**	52 913.3	4.0 %	1 110.3	
**2045**	91 265.2	4.0 %	1 075.2	
**2050**	145 615.5	4.0 %	1 046.1	

On the other hand, PHES CED value was determined by using run-of-river hydropower plant construction & decommissioning CED value. PHES was assumed to work 8 hours per day if discharged at full power rating after full charge. The operational CED value was considered zero.

Supplementary Table 26. CED values of PHES in units of MJ_{el}.

	Construction & Decommissioning	Operation	Relevant ecoinvent dataset name
	CED Value (MJ_{el} /kWh)	CED Value (MJ_{el} /kWh)	
**PHES**	19.5	N/A	hydropower plant construction, run-of-river (CA-QC)

3.4.2 Medium-term storage systems

The LCI data of A-CAES is based on Kapila et al.[87], which can run continuously for 10 hours per day from full charge to discharge at full discharge capacity. Wet and dry mass values for concrete were taken as 2,380 kg and 2,190 kg respectively [35]. The transmission network for low and medium voltage was split based on the amount of energy delivered at each voltage. In addition, the resource mix of the electricity input for construction was obtained from Statista database [88]. Given the working principle and the lack of available data, the CED value for the A-CAES operational phase was assumed to be zero.

Supplementary Table 27. CED values of A-CAES in units of MJ_{el}.

	Construction & Decommissioning	Operation	Relevant ecoinvent dataset name
	CED Value (MJ_{el} /kWh)	CED Value (MJ_{el} /kWh)	
**A-CAES, 60 MW**	178.8	N/A	market group for concrete, normal (GLO)<br>market for outside air intake, stainless steel, DN 370 (GLO)<br>market for sheet rolling steel (GLO)<br>market for steel, chromium steel 18/8 (GLO)<br>market for cast iron (GLO)<br>market for seal, natural rubber based (GLO)<br>market for wire drawing, copper (GLO)<br>market for aluminium alloy, metal matrix composite (GLO)<br>market for transmission network, electricity, medium voltage (GLO)<br>Electricity mix, 2019 (from statista)

For TES HT and TES DH, the CED value of 50 MW CSP through TES was used. The available data in the ecoinvent database[35] only provide a CED value for construction and decommissioning phases; therefore, the operational CED value was taken as zero due to the lack of data.

Supplementary Table 28. CED values of TES in units of MJ_{el}.

	Construction & Decommissioning	Operation	Relevant ecoinvent dataset name
	CED Value (MJ_{el} /kWh)	CED Value (MJ_{el} /kWh)	
**Thermal storage system, solar thermal parabolic trough, 50 MW**	327.3	N/A	market for thermal storage system, solar thermal parabolic trough, 50 MW (GLO)

3.5 Power-to-methane (e-methane) technologies

3.5.1 Electrolysis unit

LCI data of the alkaline electrolysis technology was retrieved from the appendix of the report[89], which was derived from the New Energy Externalities Development for Sustainability (NEEDS) project. The selected inventory was compared to other sources that use the same project results [90,91], and the differences between the inventories were identified and outdated data were excluded from the analysis. The raw materials required for the stack changes were embedded into the dataset for infrastructure.

Supplementary Table 29. CED values of electrolysis system in units of MJ_{el}.

	Construction & Decommissioning	Operation	Relevant ecoinvent dataset name
	CED Value (MJ_{el} /kWh)	CED Value (MJ_{el} /kWh)	
**Electrolysis unit, 930.8 kW**	4 468.1	4.99E-02	Electrolysis unit (Supplementary Note 5, Supplementary Table 40)

The report[89] states the electrolysis unit efficiency (LHV) as 62%, which is regarded as a conservative estimation. The report shows that alkaline and PEM electrolysis technologies have similar material types and quantities except that PEM electrolysis units require less space. Furthermore, the partial load behaviour of up to 20% of the nominal load was declared to be possible for both technologies. The start-up period (cold start) of both technologies was estimated to take about half an hour. Since the operating conditions and behaviour are the same, the presented LCI data can be used for both technologies.

The production capacity of the electrolysis unit is 47,250 kg H_2/year[89]. The operating load hour was assumed to be 30,000 hours, taking into account the operation with half of its capacity. These estimates were made based upon the results and the publications of the NEEDS project [91–93]. The electrolysis unit has 930.8 $kW_{el.}$ capacity, calculated using hydrogen higher heating value (39.4 kWh/kg H_2[94]), and total hydrogen production through lifetime and operating conditions. Technology progress of the alkaline electrolyser system is projected by using Böhm et al.[95] and Bogdanov et al.[4] estimations. The cost learning rates for electrolysis stack modules provided in Böhm et al.[95] was used as the energy learning rates.

Supplementary Table 30. Adjusted alkaline electrolysis system CED value according to years in units of MJ_{el}.

	Cumulative Installed Capacity	Energy Learning Rates	Construction & Decommissioning	Operation
	(GW)	%	CED Value (MJ_{el} /kWh)	CED Value (MJ_{el} /kWh)
**2015**	0	12.1 %	4 468.1	N/A
**2020**	32.8	11.5%	4 468.1	
**2025**	77.1	11.5%	3 841.6	
**2030**	198.6	11.3%	3 269.6	
**2035**	627.1	10.6%	2 772.0	
**2040**	5 008.6	10.3%	2 032.5	
**2045**	9 797.1	9.7%	1 925.5	
**2050**	15 497.1	9.4%	1 856.7	

3.5.2 DAC unit

A range of options is available for the implementation of the DAC unit in e-methane systems. It can be designed in such a way that CO_2 is captured from the atmosphere, from the flue gases of the conventional

power plants, and from exhaust gases of the industrial processes[96]. The design structure and scale of the DAC unit changes depending on its integration into the system. The integration of a DAC unit is mostly associated with further processes such as Fisher-Tropsch synthesis[97] and power-to-liquid (PtL) processes[91]. Therefore, it is difficult to find DAC life cycle inventory that addresses the right scaling and appropriate operating conditions for LUT-ESTM.

Madhu et al.[98] and Deutz and Bardow[99] focus solely on the LCA of the DAC unit, and both present reliable inventories. Both studies were selected as temperature-vacuum swing adsorbent due to recent technological improvements in the DAC unit. The life cycle inventory of the DAC unit was obtained from Deutz and Bardow[99]. The Climeworks DAC units are currently in operation in Hellisheiði (Iceland) and Hinwil (Switzerland)[99] among other sites. Hence, the obtained inventory data is addressing the real material structure of DAC units, operating conditions, and adsorbent consumption, which are also compatible with future performance targets of Climeworks.

The adsorbent consumption was assumed to be 3 kg/tCO_2[99] in line with Climeworks' future target. As the adsorbent, the composition of polyethyleneimine (PEI) and silica gel, amine on silica was preferred in view of its widespread use in the industry, as also described in Madhu et al.[98] analysis. Deutz and Bardow[99] is assumed to produce aziridine via the Wenker process, which was established as the main component of PEI. However, the chemical substance types and amounts are based on assumptions combined from various publications. Therefore, in this analysis, the adsorbent composition and its components ratios in Madhu et al.[98] were used to find an average CED value for the adsorbent consumption over the lifetime of the DAC unit.

In the LUT-ESTM model, DAC capacities are defined as MtCO_2/yr, and full load hours change depending on the region and the methanation unit operating conditions. For this reason, the CED value of the DAC unit is described as MJ_{el} /(MtCO_2/a) whereas the operational CED value is referred to as the adsorbent utilisation amount MJ_{el} /(MtCO_2/h). The maximum capacity of the DAC unit is obtained by dividing the required CO_2 amount for the methanation unit by the full load hour of the DAC unit and multiplying the result by 8760 h for one year.

Supplementary Table 31. CED values of DAC system in units of MJ_{el}.

	**Construction & Decommissioning**	**Operation**	**Relevant ecoinvent dataset name**
	**CED Value (MJ_{el} / (MtCO_2/a))**	**CED Value (MJ_{el} / (MtCO_2/h))**	
**DAC unit (from atmosphere)**	885 715.9	305.8	market group for concrete, normal (GLO)<br>market for reinforcing steel (GLO)<br>market for stone wool (GLO)<br>sheet rolling, steel (RER)<br>market for polyurethane adhesive (GLO)<br>market for wire drawing, copper (GLO)

		market for sheet rolling, aluminium (GLO)<br>market for alkyd paint, white, without solvent, in 60% solution state (RoW)<br>market for synthetic rubber (GLO)<br>ethanolamine production (RER)<br>market for spent solvent mixture (RoW)<br>market for activated silica (GLO)

3.5.3 Methanation unit

The production of methane appertains to the chemical formations, gas-liquid mass transfer transactions, and operating conditions in the reactor. For this analysis, a chemical methanation reactor was selected due to: (i) higher reaction rates, (ii) good temperature control, (iii) requiring simpler equipment, (iv) flexibility to scale the reactor size and to design multi-staged processes, and (v) having lower cost[100]. On this basis, life cycle inventory data for methane production were obtained from Zhang et al.[101], where a fixed bed reactor was designed for the PtG processes. The types of materials and nickel-based catalyst consumption quantity were estimated considering the dimension of the reactor referring to 1 Nm^3 CH_4 per hour methane formation rate, which is operated uninterruptedly for 20 years. The installed capacity of the reactor was estimated from the energy content of methane, 9.9 kW (for 97% purity)[102]. The chemical methanation reactor is also the default methanation process in the underlying LUT-ESTM technology configuration according to Bogdanov et al.[4].

Supplementary Table 32. CED values of methanation system in units of MJ_{el}.

	**Construction & Decommissioning**	**Operation**	**Relevant ecoinvent dataset name**
	**CED Value (MJ_{el} /kW)**	**CED Value (MJ_{el} /kWh)**	
**Fixed Bed Reactor, 9.9 kW**	19 283.1	8.75E-04	market for outside air intake, stainless steel, DN 370 (GLO)<br>market for steel, chromium steel 18/8 (GLO)<br>market for aluminium alloy, $AlMg_3$ (GLO)<br>market for nickel, class 1 (GLO)

3.5.4 Gas storage units (for CH_4 and H_2)

The conventional compressed air energy storage (C-CAES) LCI data was used to derive a CED value for gas storage systems. To simplify the system boundary for gas turbines, a new system boundary was identified by excluding the turbine and generators. The construction materials and quantities are given in the supplementary information[87] were adjusted. The weights of concrete[35], operating hours per day[87], electricity input components, and conversion factors[103] were assumed to be the same as in A-CAES.

The compression of air, methane, and hydrogen leads to different results regarding energy storage and content for the given volume due to the fundamentally different properties. The justified CED value for C-CAES must be adjusted in a simplified way by considering this fact. Using the Supplementary Information

provided by Kapila et al.[87], the salt cavern volume is calculated to be 210,516.9 m^3. This storage volume is assumed be the same for all cases despite their differences. The cavern capacity in kWh of the corresponding gas type is estimated based on the study of Caglayan et al.[104]. The methane density and LHV are assumed to be 0.715 kg/m^3 and 13.9 $kWh_{CH4,LHV}$/kg, respectively, while hydrogen density and LHV are taken as 0.090 kg/m^3 and 33.3 $kWh_{H2,LHV}$/kg[105].

Notably, the CED values of these storage units are not able to capture the further improvements' impacts in the process efficiencies and fuel switching during the manufacturing processes such as for the steel and aluminium sectors[106]. Tangible solid targets for these energy-intensive industries on the path to decarbonisation will have a significant impact on the required CED, especially after 2030s. Thus, the given CED values might be overestimated values considering this situation.

Supplementary Table 33. CED values of gas storage system in units of MJ_{el}.

	**Construction & Decommissioning**	**Operation**	**Relevant ecoinvent dataset name**
	**CED Value (MJ_{el} /kWh)**	**CED Value (MJ_{el} /kWh)**	
**Gas storage for CH_4, 1 kWh**	47.6	N/A	market for aluminium alloy, metal matrix composite (GLO)<br>market for cast iron (GLO)<br>market for wire drawing, copper (GLO)<br>market for steel, chromium steel 18/8 (GLO)<br>market for outside air intake, stainless steel, DN 370 (GLO)<br>market for seal, natural rubber based (GLO)<br>market for sheet rolling, steel (GLO)<br>market group for concrete, normal (GLO)<br>electricity mix, 2019 (from statista)
**Gas storage for H_2, 1 kWh**	158.2	N/A	

3.6 Fuel CED estimations

The fossil fuel CED indicates the energy demand required from the extraction, processing, and refining of fossil resources to reach the associated power plants. In this process, decline in quantity and quality of fossil resources are critical, which affect EROI results in the long term. The correlation of EROI and decay rate of fossil resource quantities have not been addressed in the clear matter, tangibly presenting the quantities and/or coefficients, even though the recent studies covers this issue[107,108]. It is open to interpretation; hence, the included assumptions may create an elusiveness in EROI analysis. Precisely because of that, our preference to estimate fossil fuel CED values based on the equation derived by Sgouridis et al.[30]. Supplementary Equation 4 is provided below.

$$EROI_{f(t)} = J + \left[EROI_{f(0)} - J\right] e^{-0.03t} \quad (4)$$

where fuel type is f, $EROI_{f(0)}$ is the EROI values in 1950, $EROI_{f(t)}$ is estimated EROI for a chosen year (t) and J is the stabilising value by 2100. The decay rate of fossil fuels, 3%, were subjected to historical

calibration and $EROI_{f(0)}$ values for natural gas, oil and coal were taken as 30, 40 and 80, respectively. On the other hand, *J* values were expected to be 10 for natural gas, 8 for oil and 20 for coal resources by 2100. These values are provided in the Supplementary material of Sgouridis et. al.[30]. The estimated values were converted to Mj_{el} /kWh by using the average efficiency of power plants, and fuel energy input to generate 1 kWh of electricity.

The technology-specific data of the PWR nuclear power plant was used to estimate the CED value of the nuclear fuel since its share of total nuclear capacity corresponds to over 80% in 2050[77,78]. The relative inventory is provided in Supplementary Table 41. All estimates related to operational aspects of LUT-ESTM and fuel CED do not contain the primary use of fossil fuel resources (such as coal, natural gas, etc.), so uranium (as resource) is not included in all calculations. However, the analysis took into account the chemical substances, other required auxiliary infrastructures as well as auxiliary fuels that must be compatible with the LUT-ESTM operating principles.

Supplementary Table 34. Fuel CED values estimated using Sgouridis et al.[30] in units of MJ_{el}.

	**Natural gas burned in CCGT**	**Coal**	**Oil and natural gas burned in OCGT**	**Nuclear**
	**Fuel CED Value (MJ_{el} /kWh)**	**Fuel CED Value (MJ_{el} /kWh)**	**Fuel CED Value (MJ_{el} /kWh)**	**Fuel CED Value (MJ_{el} /kWh)**
**2015**	0.3	0.1	0.3	0.2
**2020**	0.3	0.1	0.3	0.2
**2025**	0.3	0.1	0.3	0.2
**2030**	0.3	0.1	0.3	0.2
**2035**	0.3	0.1	0.3	0.2
**2040**	0.3	0.1	0.4	0.2
**2045**	0.3	0.2	0.4	0.2
**2050**	0.3	0.2	0.4	0.2

The central argument of EROI concentrates on the utilisation rate of fossil fuels. For this reason, we made a second estimation for oil and gas fuel CED values using the Delannoy et al.[107] analysis to foresee the deviations in EROI results. Delannoy et al.[107] follows dynamic decline functions for oil and gas resources and extends the research limits to find necessary energy requirements for the production of oil liquids; per contra, Sgouridis et al.[30] reckons EROI via the resource-curve model based on a technical approach in the line with biophysically feasible pathways for the energy transition[109]. In the second assumption, nuclear and coal fuel CED values were taken in the same as the first estimation because of insufficient data and the fact that resource type limitation (oil-focused) of Delannoy et al.[107] analysis. Note that the Sgouridis et al.[30] estimations are chosen as the default scenario in this analysis.

Supplementary Table 35. Natural gas and oil fuel CED values estimated based Delannoy et al.[107] in units of MJ_{el}.

	Natural gas burned in CCGT	Oil and natural gas burned in OCGT
	**Fuel CED Value (MJ_{el} /kWh)**	**Fuel CED Value (MJ_{el} /kWh)**
**2015**	0.2	0.4
**2020**	0.2	0.5
**2025**	0.3	0.6
**2030**	0.3	0.7
**2035**	0.4	0.9
**2040**	0.5	1.0
**2045**	0.6	1.2
**2050**	0.7	1.3

Supplementary Note 4

4 LUT-EROI Results

4.1 Global analysis

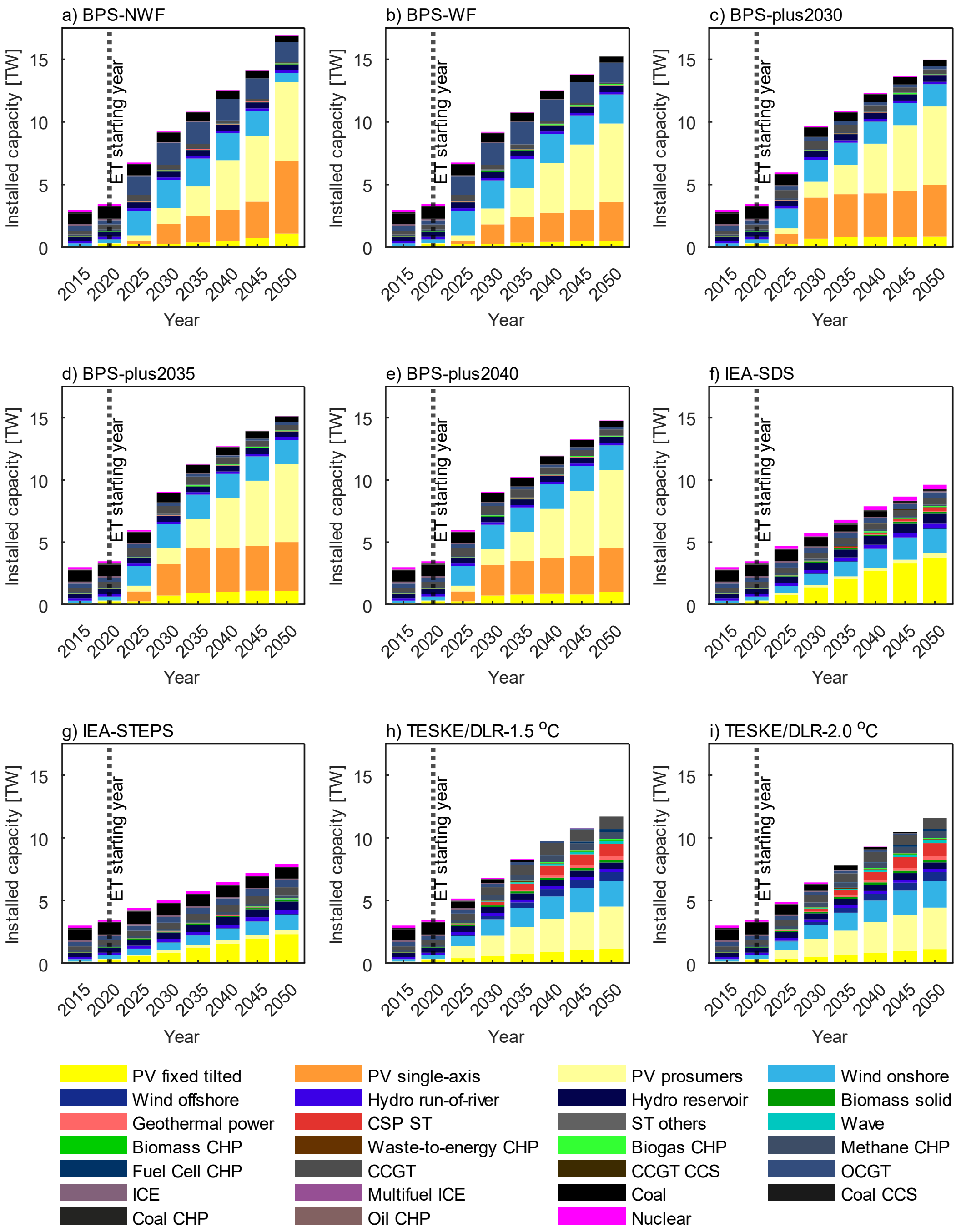

Supplementary Fig. 5. Power capacities for Global region during the energy transition period. The panels a, b, c, d, e, f show LUT scenarios, f and g show IEA scenarios, and h and i show Teske/DLR scenarios. CCGT, combined-cycle gas turbines, CCS, carbon capture and storage, CHP, combined heat and power plant, CSP, concentrated solar thermal power, ICE, internal combustion engine, OCGT, open-cycle gas turbines, PP, power plant, ST, steam turbines.

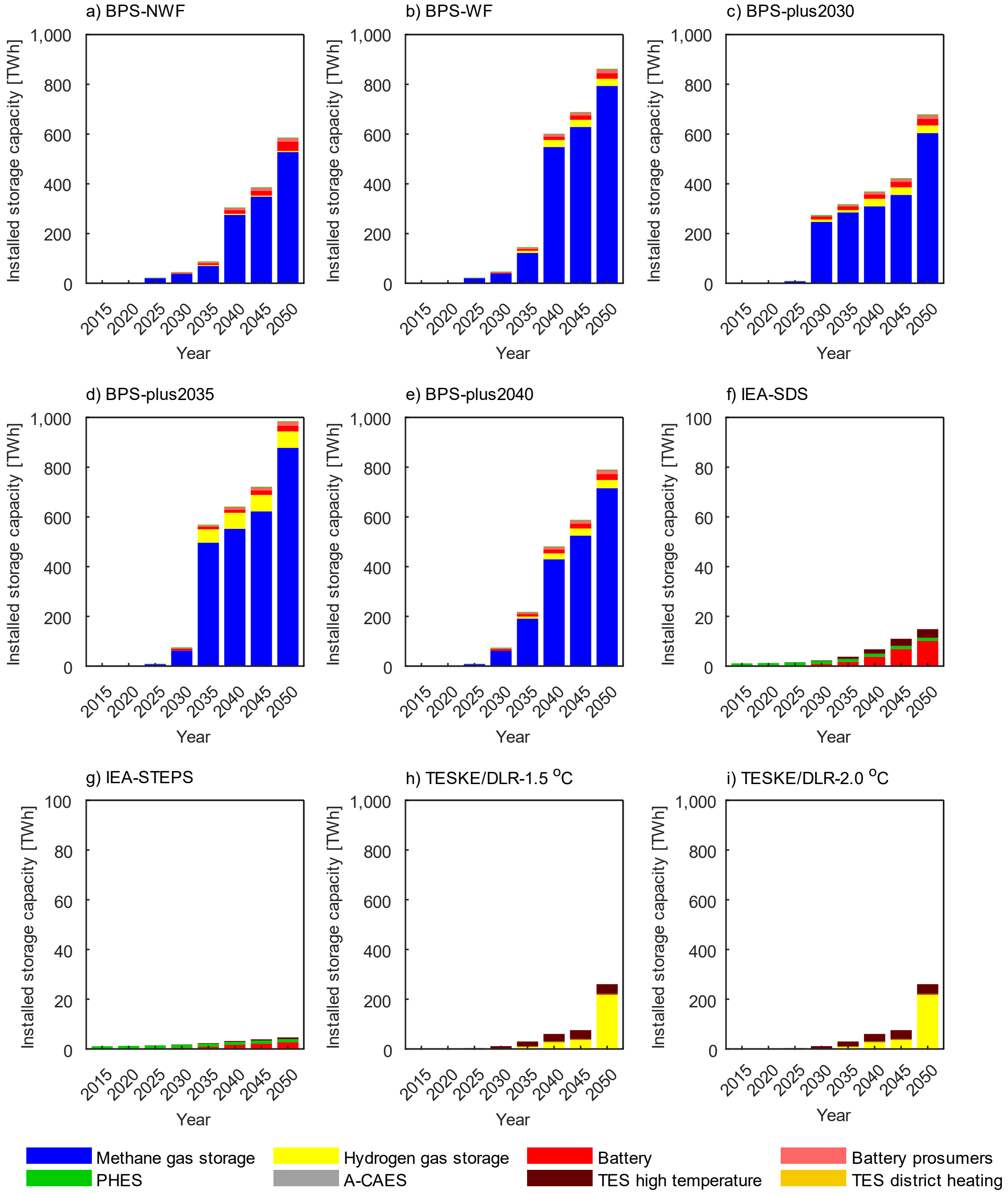

Supplementary Fig. 6. Storage capacities for Global region during energy transition. The panels a, b, c, d, e, f show LUT scenarios, f and g show IEA scenarios, and h and i show Teske/DLR scenarios. A-CAES, adiabatic compressed air storage, PHES, pumped hydro energy storage, TES DH &DH, thermal energy storage high temperature & district heating.

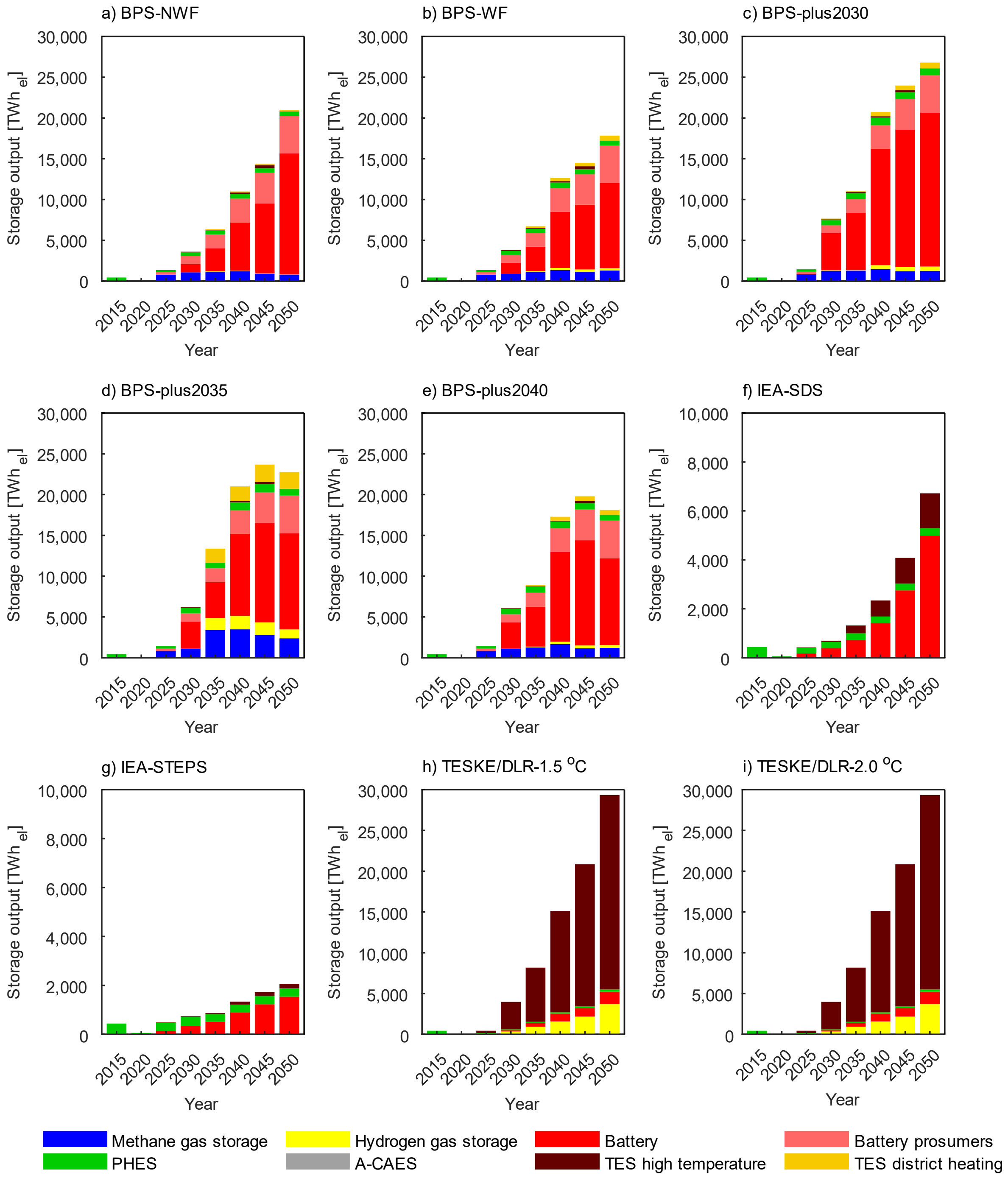

Supplementary Fig. 7. Storage throughput for Global region during energy transition. The panels a, b, c, d, e, f show LUT scenarios, f and g show IEA scenarios, and h and i show Teske/DLR scenarios. A-CAES, adiabatic compressed air storage, PHES, pumped hydro energy storage, TES DH &DH, thermal energy storage high temperature & district heating.

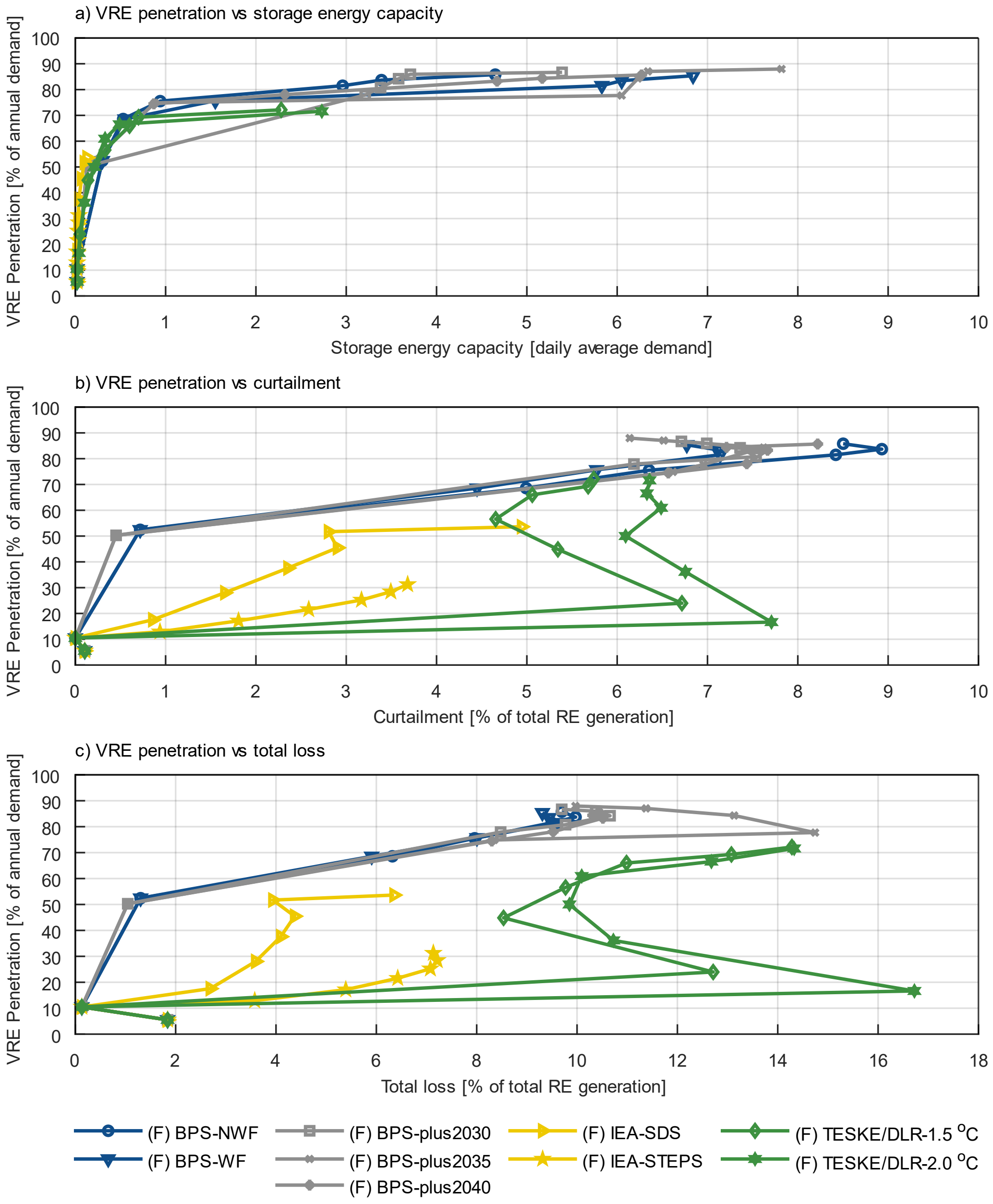

Supplementary Fig. 8. Variable renewable energy (VRE) penetration changes in the Global region. VRE penetration relationships between storage energy capacity, curtailment and total loss are shown in the panels a, b, and c. The scenarios are represented as blue lines for BPS scenarios, grey lines for BPS Plus scenarios, yellow lines for IEA scenarios and finally green lines for Teske/DLR scenarios. Note that VRE in this study refers to variable renewable energy, and it only include solar photovoltaics and wind power technologies. Renewable energy and versus are abbreviated as RE and vs, respectively.

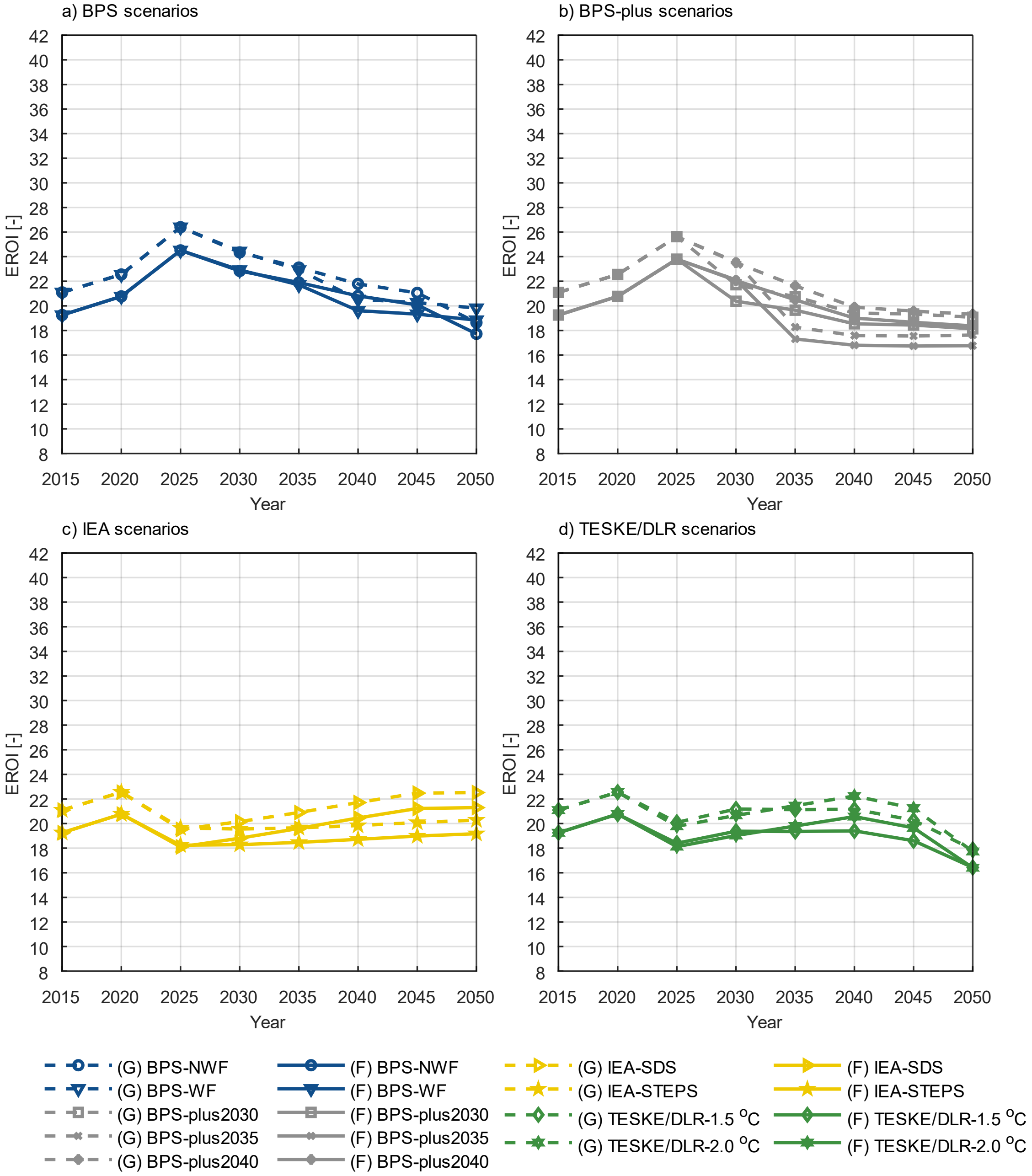

Supplementary Fig. 9. Systemwide EROI trends for Global region. The scenarios are represented as blue lines for BPS scenarios, grey lines for BPS Plus scenarios, yellow lines for IEA scenarios and finally green lines for Teske/DLR scenarios. The cut-off point for (G) EROI represents the net electricity fed to the grid (excluding transmission and distribution (T&D) losses) and for (F) EROI is the net electricity delivered to end-users (including T&D losses).

4.2 Regional analysis

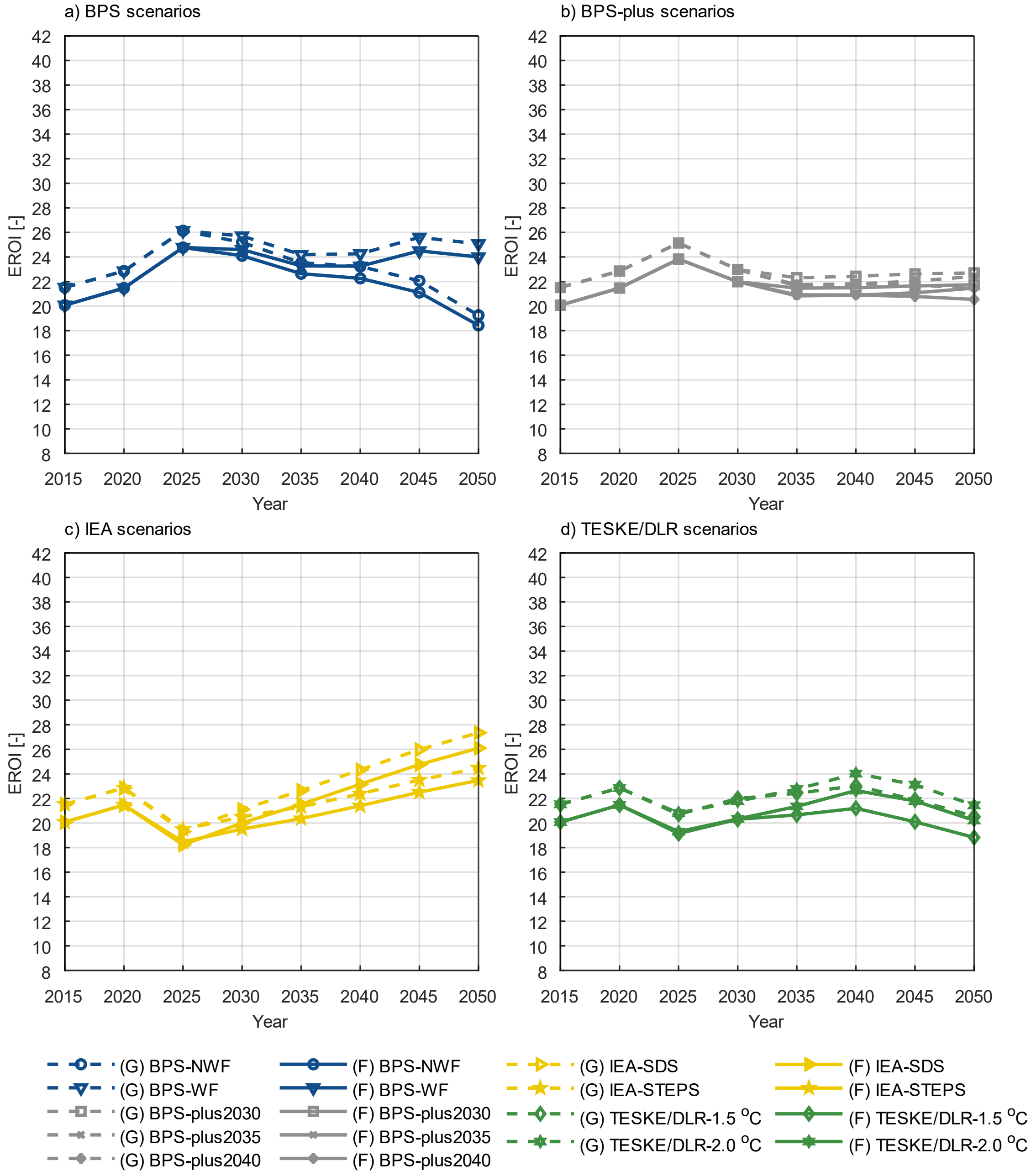

Supplementary Fig. 10. Systemwide EROI trends for Europe region. The scenarios are represented as blue lines for BPS scenarios, grey lines for BPS Plus scenarios, yellow lines for IEA scenarios and finally green lines for Teske/DLR scenarios. The cut-off point for (G) EROI represents the net electricity fed to the grid (excluding transmission and distribution (T&D) losses) and for (F) EROI is the net electricity delivered to end-users (including T&D losses).

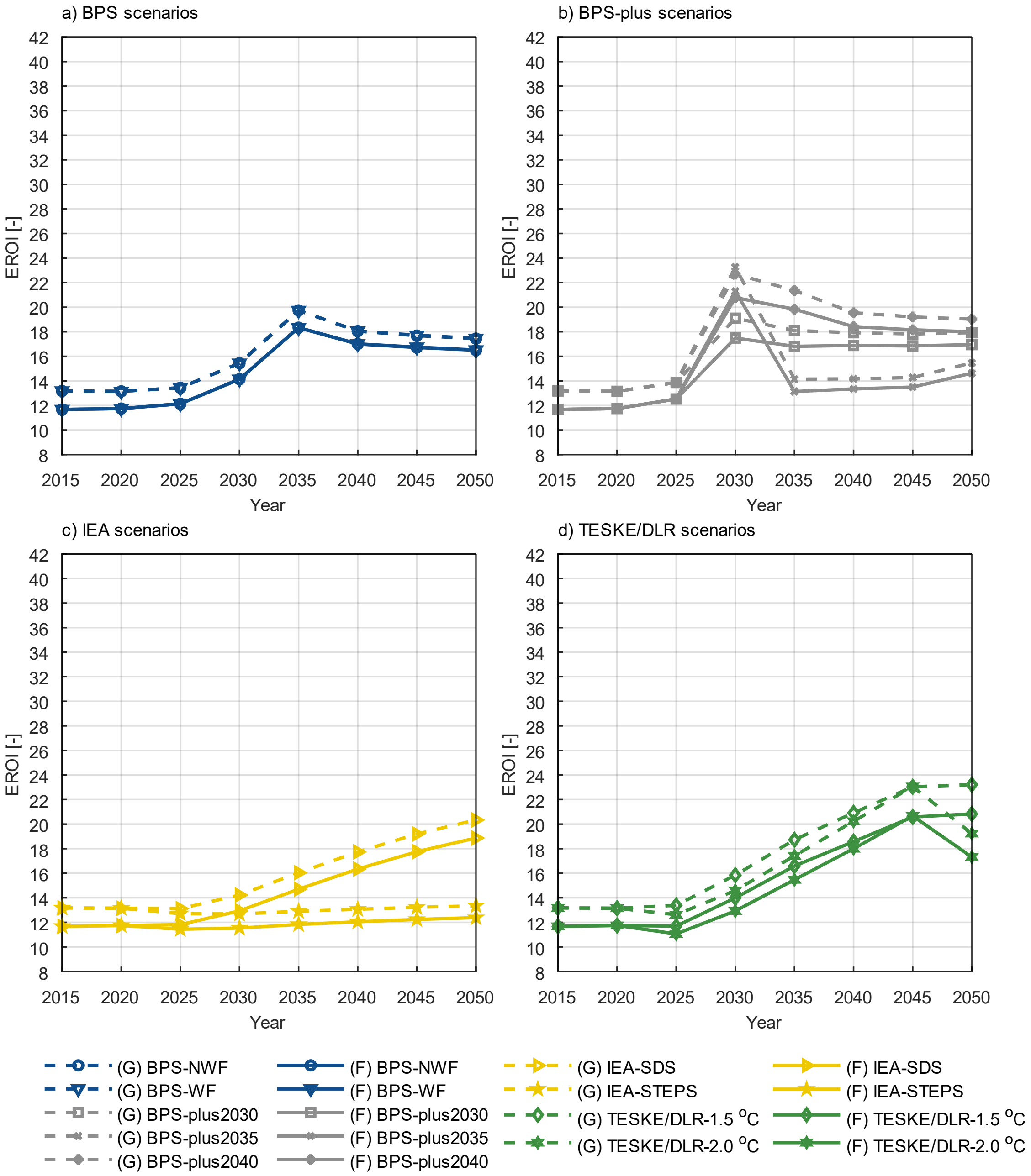

Supplementary Fig. 11. Systemwide EROI trends for Middle East and North Africa region. The scenarios are represented as blue lines for BPS scenarios, grey lines for BPS Plus scenarios, yellow lines for IEA scenarios and finally green lines for Teske/DLR scenarios. The cut-off point for (G) EROI represents the net electricity fed to the grid (excluding transmission and distribution (T&D) losses) and for (F) EROI is the net electricity delivered to end-users (including T&D losses).

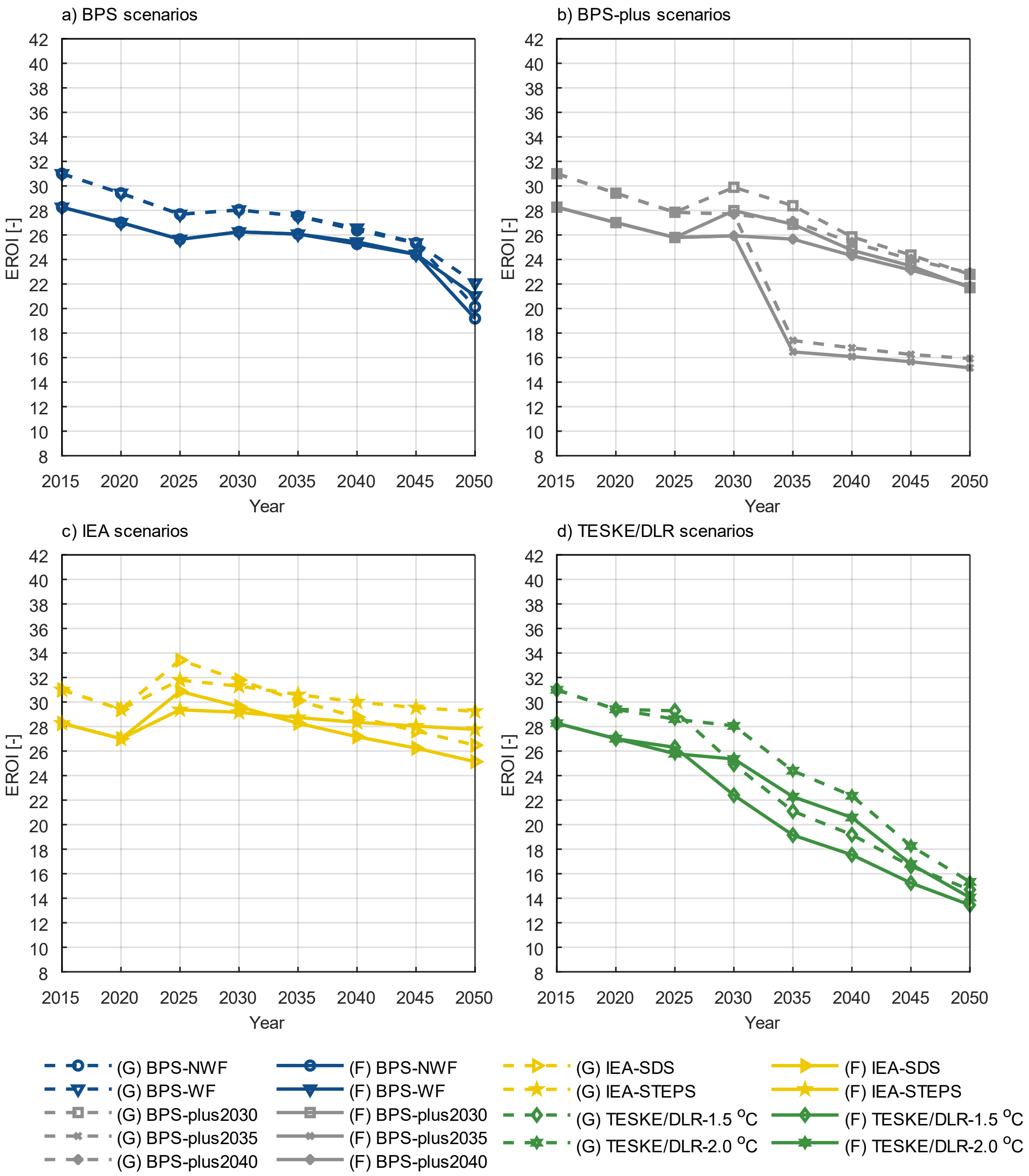

Supplementary Fig. 12. Systemwide EROI trends for Eurasia region. The scenarios are represented as blue lines for BPS scenarios, grey lines for BPS Plus scenarios, yellow lines for IEA scenarios and finally green lines for Teske/DLR scenarios. The cut-off point for (G) EROI represents the net electricity fed to the grid (excluding transmission and distribution (T&D) losses) and for (F) EROI is the net electricity delivered to end-users (including T&D losses).

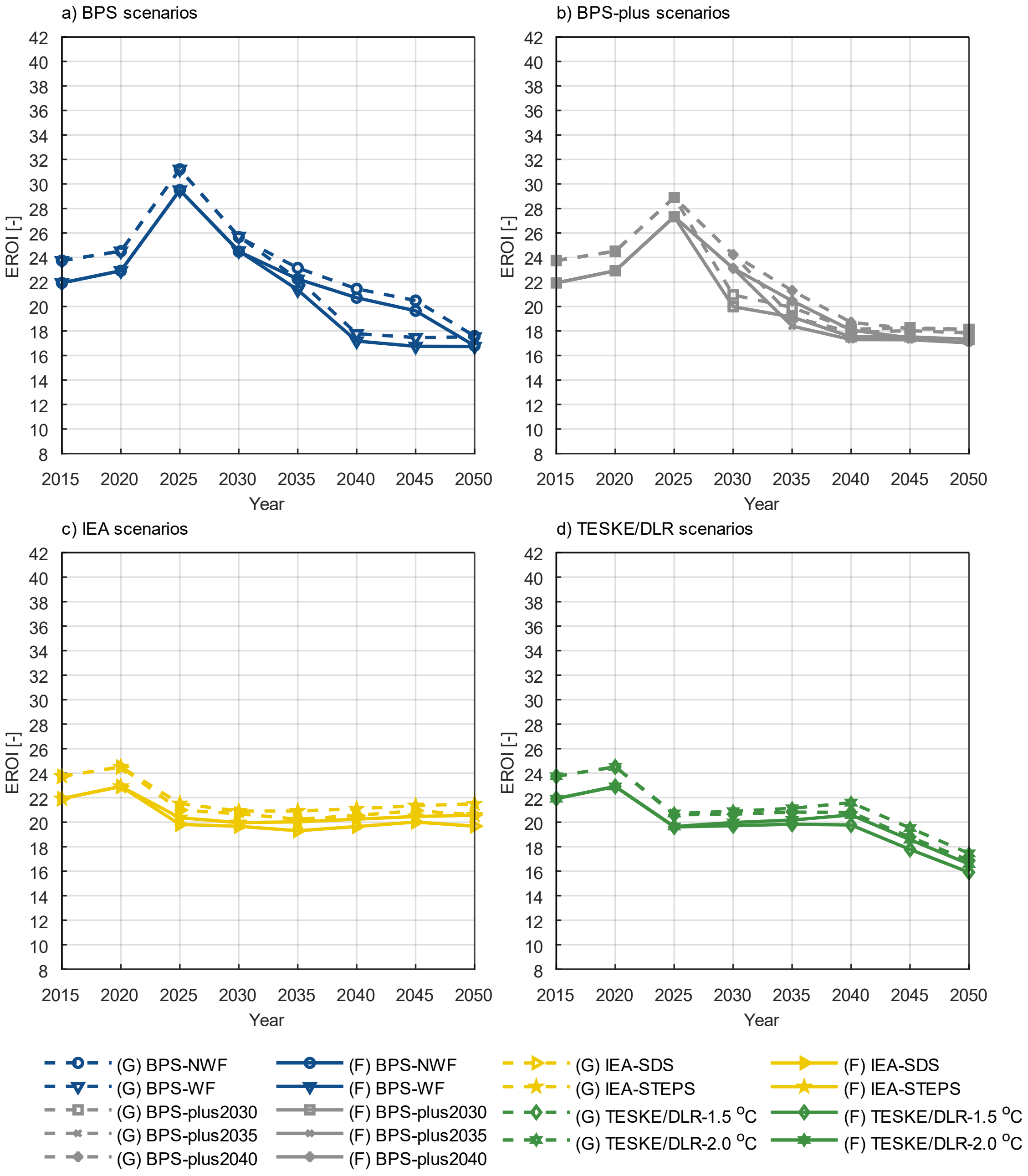

Supplementary Fig. 13. Systemwide EROI trends for Northeast Asia region. The scenarios are represented as blue lines for BPS scenarios, grey lines for BPS Plus scenarios, yellow lines for IEA scenarios and finally green lines for Teske/DLR scenarios. The cut-off point for (G) EROI represents the net electricity fed to the grid (excluding transmission and distribution (T&D) losses) and for (F) EROI is the net electricity delivered to end-users (including T&D losses).

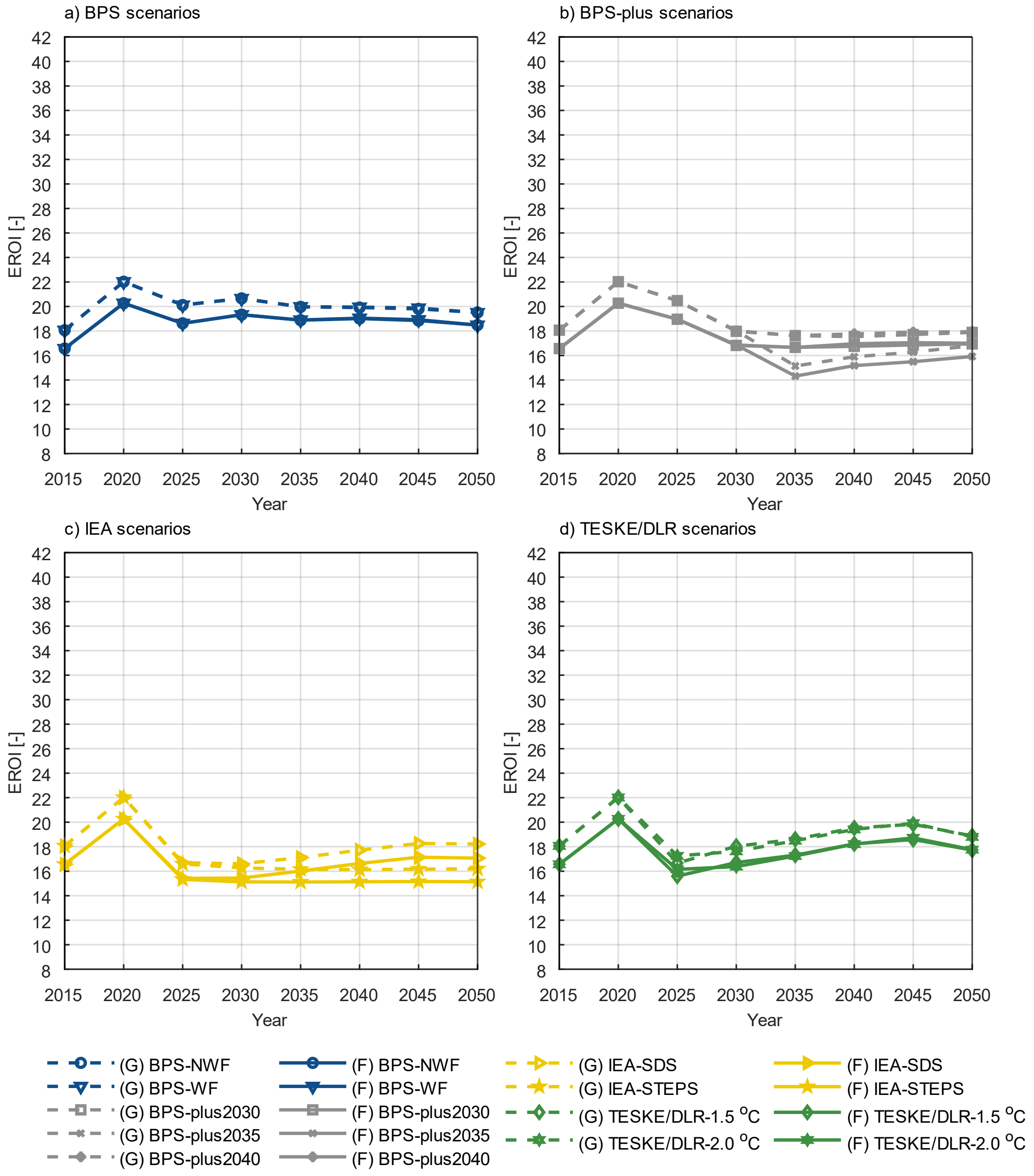

Supplementary Fig. 14. Systemwide EROI trends for Southeast Asia region. The scenarios are represented as blue lines for BPS scenarios, grey lines for BPS Plus scenarios, yellow lines for IEA scenarios and finally green lines for Teske/DLR scenarios. The cut-off point for (G) EROI represents the net electricity fed to the grid (excluding transmission and distribution (T&D) losses) and for (F) EROI is the net electricity delivered to end-users (including T&D losses).

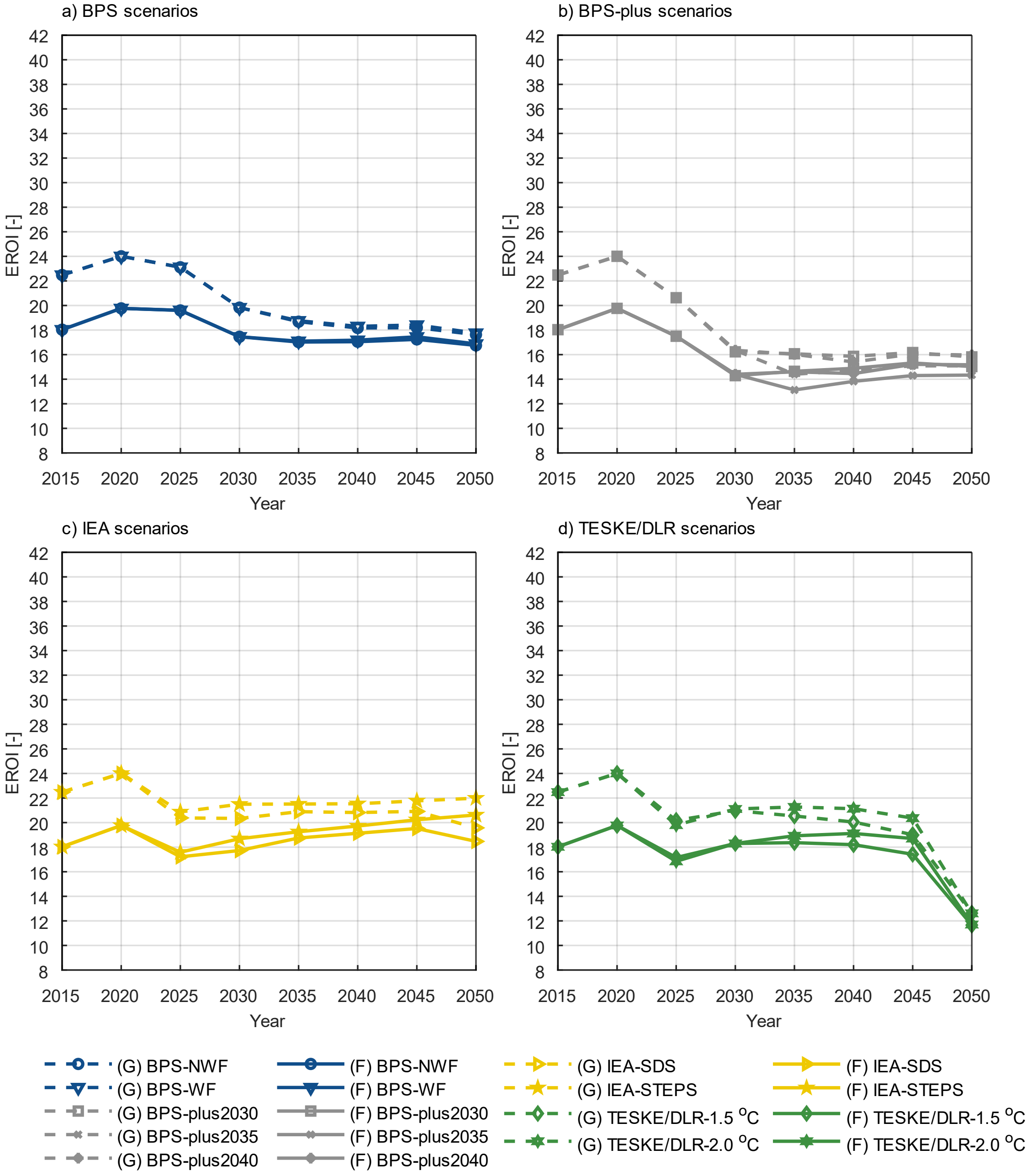

Supplementary Fig. 15. Systemwide EROI trends for South Asian Association for Regional Cooperation region. The scenarios are represented as blue lines for BPS scenarios, grey lines for BPS Plus scenarios, yellow lines for IEA scenarios and finally green lines for Teske/DLR scenarios. The cut-off point for (G) EROI represents the net electricity fed to the grid (excluding transmission and distribution (T&D) losses) and for (F) EROI is the net electricity delivered to end-users (including T&D losses).

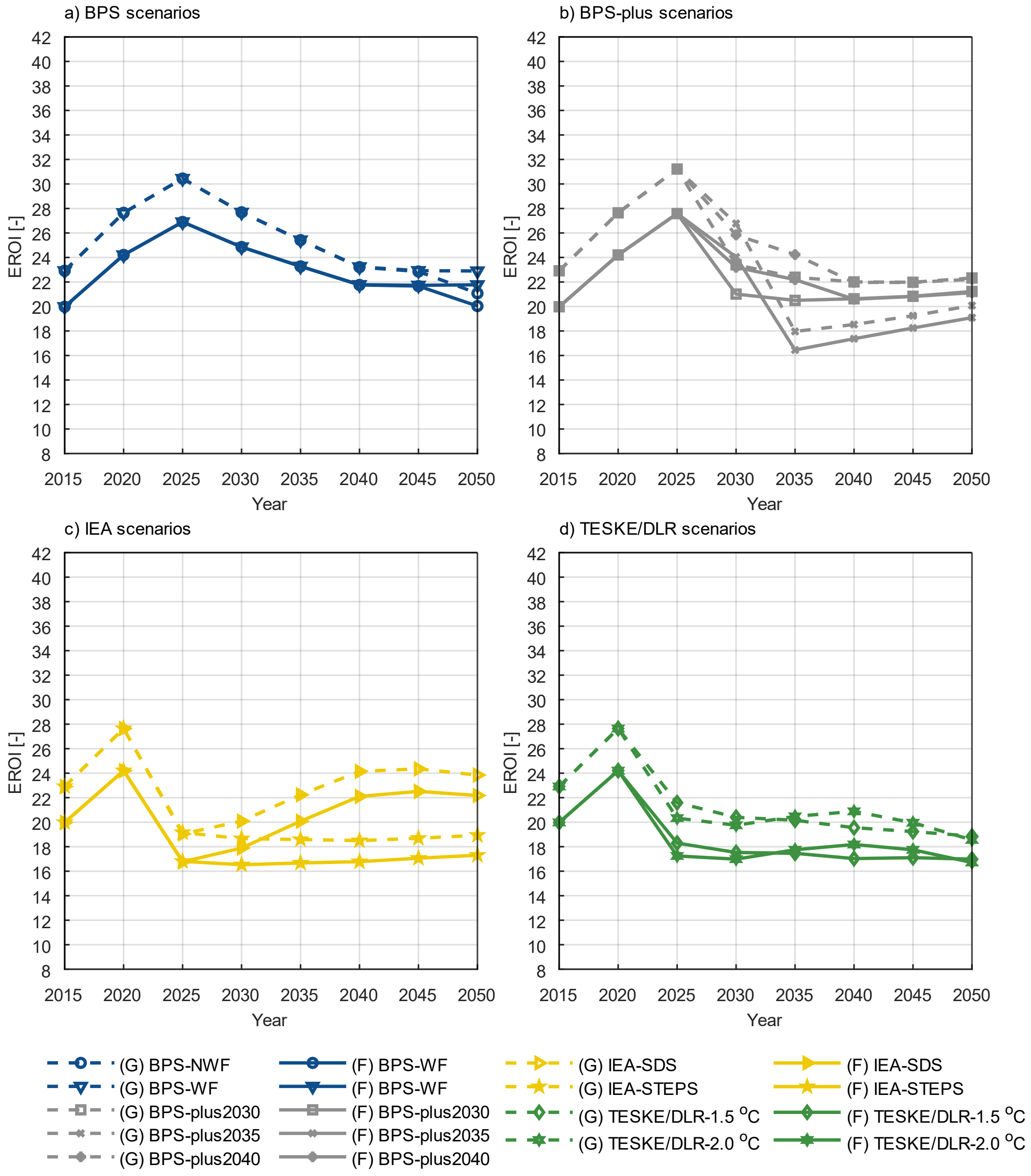

Supplementary Fig. 16. Systemwide EROI trends for Sub-Saharan Africa region. The scenarios are represented as blue lines for BPS scenarios, grey lines for BPS Plus scenarios, yellow lines for IEA scenarios and finally green lines for Teske/DLR scenarios. The cut-off point for (G) EROI represents the net electricity fed to the grid (excluding transmission and distribution (T&D) losses) and for (F) EROI is the net electricity delivered to end-users (including T&D losses).

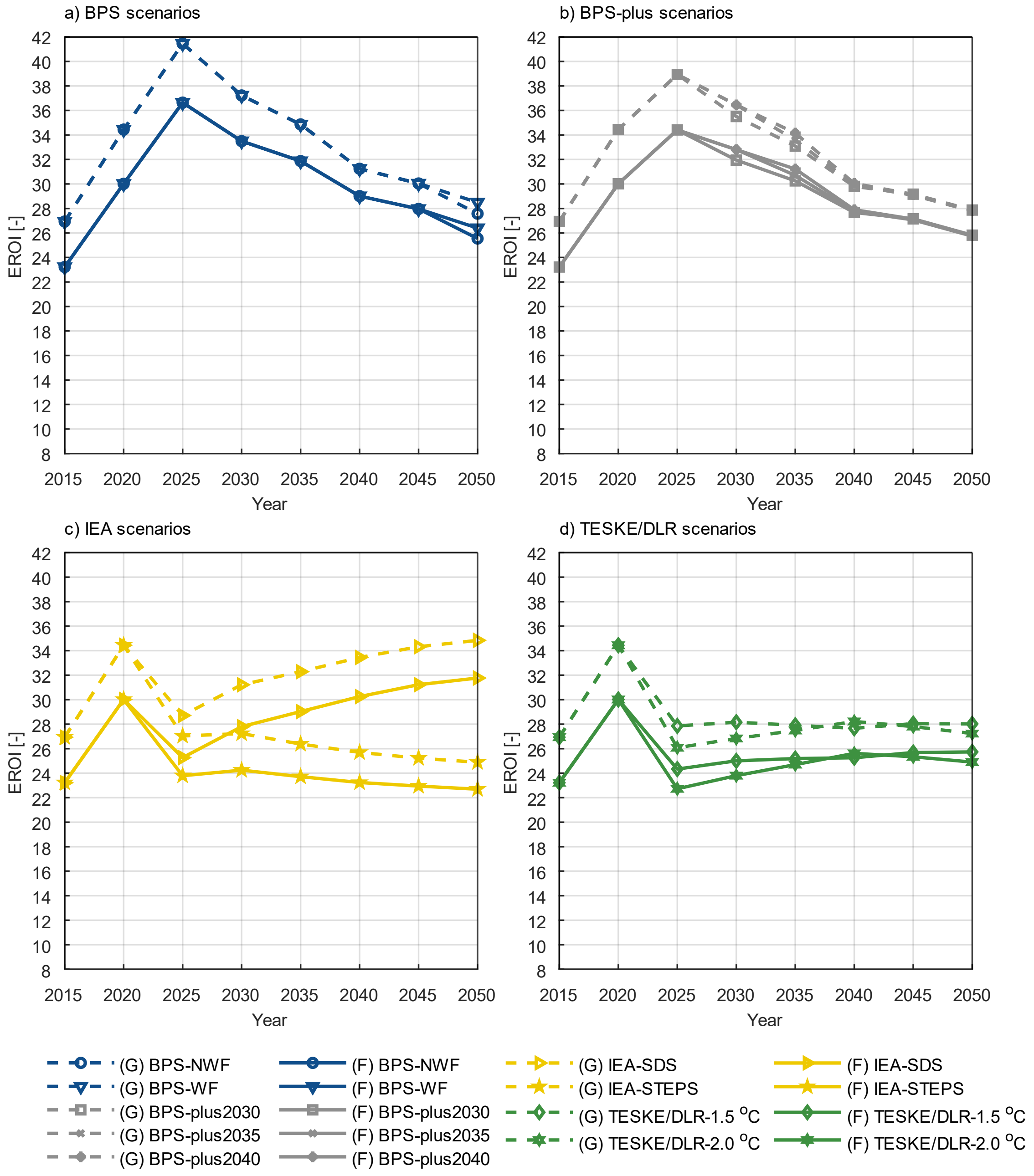

Supplementary Fig. 17. Systemwide EROI trends for South America region. The scenarios are represented as blue lines for BPS scenarios, grey lines for BPS Plus scenarios, yellow lines for IEA scenarios and finally green lines for Teske/DLR scenarios. The cut-off point for (G) EROI represents the net electricity fed to the grid (excluding transmission and distribution (T&D) losses) and for (F) EROI is the net electricity delivered to end-users (including T&D losses).

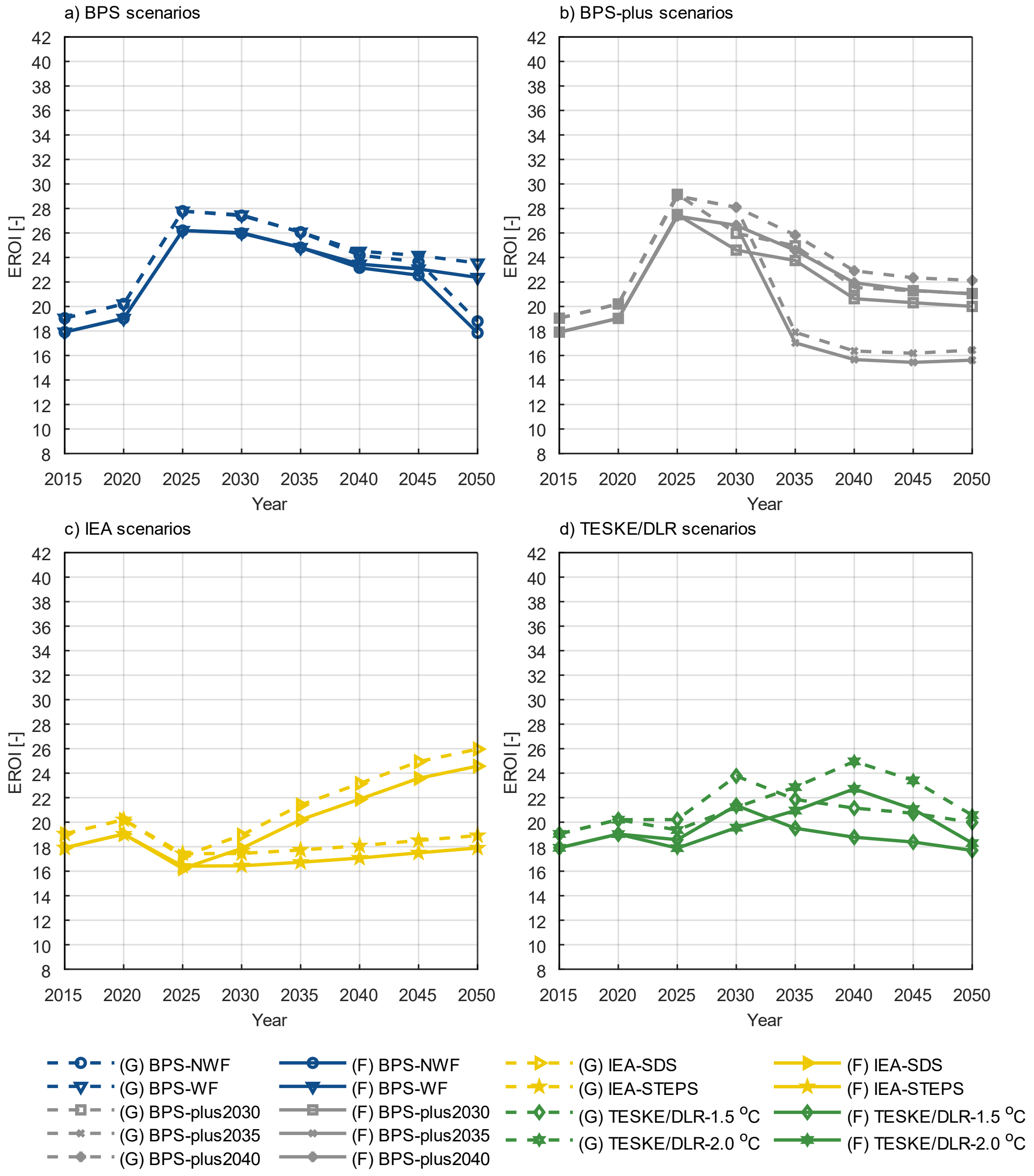

Supplementary Fig. 18. Systemwide EROI trends for North America region. The scenarios are represented as blue lines for BPS scenarios, grey lines for BPS Plus scenarios, yellow lines for IEA scenarios and finally green lines for Teske/DLR scenarios. The cut-off point for (G) EROI represents the net electricity fed to the grid (excluding transmission and distribution (T&D) losses) and for (F) EROI is the net electricity delivered to end-users (including T&D losses).

Supplementary Note 5

5 Inventory for Specific Technologies

Supplementary Table 36. Life cycle inventory of biomass CHP plant.

	Dataset
**Stainless steel (304)**	market for electric arc furnace converter (GLO)<br>market group for electricity, high voltage (GLO)<br>market for scrap steel (RoW)<br>market for iron ore concentrate (GLO)<br>market for ferrochromium, high-carbon, 68% Cr (GLO)<br>market for ferronickel (GLO)<br>market for molybdenum (GLO)
**Stainless steel (316)**	market for electric arc furnace converter (GLO)<br>market group for electricity, high voltage (GLO)<br>market for scrap steel (RoW)<br>market for iron ore concentrate (GLO)<br>market for ferrochromium, high-carbon, 68% Cr (GLO)<br>market for ferronickel (GLO)<br>market for molybdenum (GLO)
**Outside enclosure**	stainless steel (316)<br>paint production, for electrostatic painting for aluminium (GLO)
**Biomass silo**	reinforcing steel production (RoW)<br>stainless steel (316)<br>paint production, for electrostatic painting for aluminium (GLO)<br>market for copper, cathode (GLO)
**Feed hopper & valves**	stainless steel (316)<br>reinforcing steel production (RoW)<br>market for copper, cathode (GLO)<br>market for synthetic rubber (GLO)<br>market for cast iron (GLO)<br>market for zinc (GLO)
**Pre-burner**	stainless steel (304)<br>stainless steel (316)<br>market for silicon carbide (GLO)<br>market for copper, cathode (GLO)<br>market for ceramic tile (GLO)<br>market for synthetic rubber (GLO)
**Gasifier (including ash disposal unit)**	stainless steel (316)<br>market for stone wool (GLO)<br>reinforcing steel production (RoW)<br>market for copper, cathode (GLO)
**Scrubber**	stainless steel (304)<br>stainless steel (316)

	market for aluminium, primary, ingot (RoW)<br>market for magnetite (GLO)<br>market for copper, cathode (GLO)<br>market for tetrafluoroethylene (GLO)<br>market for aluminium, wrought alloy (GLO)<br>tempering, flat glass (RoW)
**Pumps and blower**	stainless steel (304)<br>stainless steel (316)<br>market for tube insulation, elastomer (GLO)<br>market for cast iron (GLO)<br>reinforcing steel production (RoW)<br>market for aluminium, primary, ingot (RoW)<br>market for lubricating oil (RER)<br>market for polyurethane, flexible foam (RoW)
**Aftercooler & demister**	stainless steel (316)<br>market for cast iron (GLO)<br>reinforcing steel production (RoW)<br>market for brass (RoW)<br>market for aluminium, primary, ingot (RoW)<br>market for ceramic tile (GLO)<br>paint production, for electrostatic painting for aluminium (GLO)
**Solvent handling**	stainless steel (316)<br>market for graphite (GLO)<br>market for thermoforming of plastic sheets (GLO)<br>market for polypropylene, granulate (GLO)<br>market for tube insulation, elastomere (GLO)<br>market for tetrafluoroethylene (GLO)<br>reinforcing steel production (RoW)<br>market for synthetic rubber (GLO)<br>paint production, for electrostatic painting for aluminium (GLO)<br>market for polyester resin, unsaturated (RoW)<br>market for copper, cathode (GLO)<br>market for polyethylene terephthalate, granulate, amorphous (GLO)
**Heat exchanger (including pipes)**	paint production, for electrostatic painting for aluminium (GLO)<br>market for polyester resin, unsaturated (RoW)<br>market for tetrafluoroethylene (GLO)<br>market for synthetic rubber (GLO)<br>market for polyethylene terephthalate, granulate, amorphous (GLO)<br>market for brass (RoW)<br>market for printed wiring board, through-hole mounted, unspecified, Pb free (GLO)<br>market for aluminium, primary, ingot (RoW)<br>market for copper, cathode (GLO)<br>reinforcing steel production (RoW)
**Outside the skid**	market for aluminium, primary, ingot (RoW)

	market for silicon carbide (GLO)<br>market for graphite (GLO)<br>market for synthetic rubber (GLO)<br>reinforcing steel production (RoW)<br>market for cast iron (GLO)<br>market for iron-nickel-chromium alloy (GLO)<br>market for glass fibre reinforced plastic, polyamide, injection moulded (GLO)<br>market for tetrafluoroethylene (GLO)
**Gas engine, 250 kW**	stainless steel (304)<br>reinforcing steel production (RoW)<br>market for steel, low-alloyed (GLO)<br>market for copper, cathode (GLO)<br>market for aluminium, primary, ingot (RoW)<br>market for iron-nickel-chromium alloy (GLO)<br>market for polyethylene, high density, granulate (GLO)<br>market for polyvinylchloride, suspension polymerised (GLO)
**Additional materials for chp unit, transportation of willow, and chemical materials for willow**	wood chipping, industrial residual wood, stationary electric chipper (GLO)<br>market for lubricating oil (RoW)<br>market for diesel, burned in agricultural machinery (GLO)<br>market for lime (RoW)<br>market for glyphosate (GLO)<br>market for organic nitrogen fertiliser, as N (GLO)<br>market for single superphosphate (RoW)<br>market for organo-mineral potassium fertiliser (GLO)

Supplementary Table 37. Life cycle inventory of CCS unit.

	Dataset
**CO_2 capture infrastructure**	market for steel, chromium steel 18/8, hot rolled (GLO)<br>market group for concrete, normal (GLO)<br>market for transport, freight, lorry 16-32 metric ton, EURO6 (RoW)
**CO_2 compressor infrastructure**	market group for concrete, normal (GLO)<br>market for steel, low-alloyed (GLO)<br>market for copper, cathode (GLO)<br>market for polyethylene, high density, granulate (GLO)<br>market for electricity, medium voltage (CH)
**CO_2 pipeline infrastructure**	market for sand (RoW)<br>market for reinforcing steel (GLO)<br>market for drawing of pipe, steel (GLO)<br>market for polyethylene, high density, granulate (GLO)<br>market for transport, freight, lorry 16-32 metric ton, EURO6 (RoW)<br>market for diesel, burned in building machine (GLO)<br>market for inert waste, for final disposal (RoW)
**CO_2 injection facility**	deep well drilling, for geothermal power, onshore, 6000m (GLO)<br>market for sand (RoW)<br>market for steel, low-alloyed (GLO)<br>market for steel, chromium steel 18/8, hot rolled (GLO)<br>market group for concrete, normal (GLO)<br>market for transport, freight, lorry 16-32 metric ton, EURO6 (RoW)<br>market for wire drawing, copper (GLO)
**MEA chemical plant infrastructure**	market for steel, low-alloyed (GLO)<br>market for steel, chromium steel 18/8, hot rolled (GLO)<br>market for brazing solder, cadmium free (GLO)<br>market for stone wool (GLO)<br>market for concrete, sole plate and foundation (CH)<br>market for electronics, for control units (GLO)<br>market for electricity, medium voltage (CH)<br>market for transport, freight, lorry 16-32 metric ton, EURO6 (RoW)
**MEA production**	market for ethylene oxide (RoW)<br>market for ammonia, anhydrous, liquid (RoW)<br>market group for electricity, medium voltage (GLO)<br>market for heat, district or industrial, natural gas (RoW)<br>market for transport, freight, lorry 16-32 metric ton, EURO6 (RoW)
**NaOH**	sodium hydroxide to generic market for neutralising agent (GLO)
**Activated carbon**	market for activated carbon, granular (GLO)

Supplementary Table 38. Life cycle inventory of nuclear power plants.

	Dataset
SMR	market for steel, low-alloyed (GLO)
	market group for concrete, normal (GLO)
	reinforcing steel production (RoW)
AHWR	market for aluminium, primary, ingot (RoW)
	market for steel, chromium steel 18/8 (GLO)
	market for copper, cathode (GLO)
	treatment of waste concrete, inert material landfill (RoW)
	market for drawing of pipe, steel (GLO)
	graphite production (RoW)
	market for steel, low-alloyed, hot rolled (GLO)
	polycarbonate production (RoW)
	market for polyester resin, unsaturated (RoW)
	market for polyethylene terephthalate, granulate, amorphous (GLO)
	polymethyl methacrylate production, beads (RoW)
	market for polyvinylchloride, suspension polymerised (GLO)
	reinforcing steel production (RoW)
	market for sheet rolling, aluminium (GLO)
	market for sheet rolling, chromium steel (GLO)
	market for sheet rolling, steel (GLO)
	market for steel, low-alloyed (GLO)
	market for synthetic rubber (GLO)
	market for tin (GLO)
	market for wire drawing, copper (GLO)
	market group for electricity, medium voltage (GLO)
	market for welding, arc, steel (GLO)
	market group for concrete, normal (GLO)
	market for excavation, hydraulic digger (GLO)
	market for excavation, skid-steer loader (GLO)
	sawn wood production, hardwood, dried (u=10%), planed (RoW)
	market for diesel, burned in building machine (GLO)
	heat production, light fuel oil, at industrial furnace 1MW (RoW)
	market for transport, freight train (RoW)
	market for transport, freight, lorry >32 metric ton, EURO5 (RoW)
	market for transport, freight, lorry 16-32 metric ton, EURO5 (RoW)
GT-MHR	market for aluminum, primary, ingot (RoW)
	market for steel, chromium steel 18/8 (GLO)
	market for copper, cathode (GLO)
	treatment of waste concrete, inert material landfill (RoW)
	market for drawing of pipe, steel (GLO)
	graphite production (RoW)
	market for steel, low-alloyed, hot rolled (GLO)
	polycarbonate production (RoW)
	market for polyester resin, unsaturated (RoW)

	market for polyethylene terephthalate, granulate, amorphous (GLO)
	polymethyl methacrylate production, beads (GLO)
	market for polyvinylchloride, suspension polymerised (RoW)
	reinforcing steel production (RoW)
	market for sheet rolling, aluminum (GLO)
	market for sheet rolling, chromium steel (GLO)
	market for sheet rolling, steel (GLO)
	market for steel, low-alloyed (GLO)
	market for synthetic rubber (GLO)
	market for tin (GLO)
	market for wire drawing, copper (GLO)
	market group for electricity, medium voltage (GLO)
	market for welding, arc, steel (GLO)
	market group for concrete, normal (GLO)
	market for excavation, hydraulic digger (GLO)
	market for excavation, skid-steer loader (GLO)
	sawn wood production, hardwood, dried (u=10%), planed (RoW)
	market for diesel, burned in building machine (GLO)
	heat production, light fuel oil, at industrial furnace 1MW (RoW)
	market for transport, freight train (RoW)
	market for transport, freight, lorry >32 metric ton, EURO5 (RoW)
	market for transport, freight, lorry 16-32 metric ton, EURO5 (RoW)

Supplementary Table 39. LCI for operation CED value for PWR nuclear power plant.

	**Dataset**
**Materials used in the operational phase of PWR nuclear power plant, 1000 MW**	market for acetylene (RoW)
	market for argon, liquid (RoW)
	market for boric acid, anhydrous, powder (GLO)
	market for carbon dioxide, liquid (RoW)
	market for chemicals, inorganic (GLO)
	market for chemical, organic (GLO)
	market for diesel, burned in diesel-electric generating set, 10MW (GLO)
	market for hydrogen, liquid (RoW)
	market for lubricating oil (RoW)
	market for nitrogen, liquid (RoW)
	market for oxygen, liquid (RoW)
	market for sodium hypochlorite, without water, in 15% solution state (RoW)
	market for water, decarbonised (RoW)
	market for fly ash and scrubber sludge (RoW)
	market for hazardous waste, for incineration (RoW)
	market for low level radioactive waste (GLO)
	market for low level radioactive waste for final repository (GLO)
	market for spent nuclear fuel (GLO)
	market for waste mineral oil (RoW)

Supplementary Table 40. Life cycle inventory of electrolysis unit components.

	**Dataset**
**Infrastructure**	market for steel, chromium steel 18/8 (GLO)
	market for nickel, class 1 (GLO)
	market for synthetic rubber (GLO)
	market for reinforcing steel (GLO)
	market for copper, cathode (GLO)
	market for tube insulation, elastomer (GLO)
	market for aluminium, primary, liquid (GLO)
	market for acrylonitrile-butadiene-styrene copolymer (GLO)
	market for polyethylene, low density, granulate (GLO)
	market for glass fibre (GLO)
	market for cast iron (GLO)
	market for nylon 6-6, glass filled (RER)
	market for transport, freight, lorry 16-32 metric tonne, EURO6 (RER)
**Membrane compressor**	market for reinforcing steel (GLO)
	market for steel, chromium steel 18/8 (GLO)
	market for cast iron (GLO)
	market for ethylene glycol (GLO)
	market for lubricating oil (RER)
	market for aluminium, primary, liquid (GLO)
	market for tube insulation, elastomer (GLO)
	market for copper, cathode (GLO)
	market group for electricity, high voltage (GLO)
	heat production, natural gas, at industrial furnace >100kW (Europe without CH)
	market for transport, freight, lorry 16-32 metric tonne, EURO6 (RER)
**Storage module**	market for steel, chromium steel 18/8 (GLO)
	market group for electricity, high voltage (GLO)
	market for diesel, burned in building machine (GLO)
	market for transport, freight, lorry 16-32 metric tonne, EURO6 (RER)
**Walls & foundation**	market for reinforcing steel (GLO)
	market for flat glass, coated (RER)
	market for gypsum fibreboard (GLO)
	market for silica sand (GLO)
	market group for concrete, normal (GLO)
	market for concrete, high exacting requirements (CH)
	market for gravel, crushed (CH)
	market for lubricating oil (RER)
	market group for electricity, high voltage (GLO)
	market for diesel, burned in building machine (GLO)
	market for transport, freight, lorry 16-32 metric tonne, EURO6 (RER)
**Other components**	market for reinforcing steel (GLO)
	market for nitrogen, liquid (RER)
	market for steel, chromium steel 18/8 (GLO)
	market for polypropylene, granulate (GLO)

	market for transport, freight, lorry 16-32 metric tonne, EURO6 (RER)
**Maintenance**	market for lubricating oil (RER)<br>market group for electricity, high voltage (GLO)<br>heat production, natural gas, at industrial furnace >100kW (Europe without CH)<br>market for transport, freight, lorry 16-32 metric tonne, EURO6 (RER)

Supplementary Table 41. LCI for estimating fuel CED value for nuclear power plant.

	Dataset
**Uranium mine operation, underground**	market for blasting (GLO)
	market for diesel, burned in diesel-electric generating set, 10MW (GLO)
	market for mine infrastructure, underground, uranium (GLO)
	market group for transport, freight train (GLO)
	market group for transport, freight, inland waterways, barge (GLO)
	market group for transport, freight, lorry, unspecified (GLO)
**Uranium production, in yellowcake**	market for diesel, burned in diesel-electric generating set, 10MW (GLO)
	market for steel, chromium steel 18/8 (GLO)
	market for sulfuric acid (RoW)
	market for water, decarbonised (RoW)
	Uranium mine operation, underground (calculated)
**Uranium hexafluoride production**	market for ammonia, anhydrous, liquid (RoW)
	market for cement, unspecified (RoW)
	market for chemicals, inorganic (GLO)
	market for chemical, organic (GLO)
	market group for electricity, medium voltage (GLO)
	market group for heat, district or industrial, natural gas (GLO)
	market for hydrogen fluoride (RoW)
	market for nitric acid, without water, in 50% solution state (RER without RU)
	market for quicklime, milled, loose (RoW)
	market for uranium conversion facility (GLO)
	Uranium production, in yellowcake (calculated)
	market for water, decarbonised (RoW)
	market for average incineration residue (RoW)
	market for low level radioactive waste (GLO)
**Uranium, enriched 3.8% per separative work unit**	market for acetylene (RoW)
	market for aluminium, wrought alloy (GLO)
	market for argon, liquid (RoW)
	market for brass (RoW)
	market for chemicals, inorganic (GLO)
	market for chemical, organic (GLO)
	market for concrete, normal (RoW)
	market for diesel, burned in diesel-electric generating set, 10MW (GLO)
	market group for electricity, high voltage (GLO)
	market group for electricity, medium voltage (GLO)
	market for heat, district or industrial, natural gas (RoW)
	market for hydrochloric acid, without water, in 30% solution state (RoW)
	market for hydrogen peroxide, without water, in 50% solution state (RoW)
	market for hydrogen, liquid (RoW)
	market for lubricating oil (RoW)
	market for methanol (GLO)
	market for nitric acid, without water, in 50% solution state (RER without RU)
	market for nitrogen, liquid (RoW)

	market for oxygen, liquid (RoW)<br>market for phosphoric acid, fertiliser grade, without water, in 70% solution state (RoW)<br>market for polyvinylchloride, bulk polymerised (GLO)<br>market for soap (GLO)<br>market for sodium hydroxide, without water, in 50% solution state (GLO)<br>market for steel, low-alloyed, hot rolled (GLO)<br>market for uranium enrichment centrifuge facility (GLO)<br>market for uranium enrichment diffusion facility (GLO)<br>Uranium hexafluoride production (calculated)<br>market for water, decarbonised (RoW)<br>market for low level radioactive waste (GLO)<br>market for municipal solid waste (RoW)<br>market for spent anion exchange resin from potable water production (RoW)<br>market for waste mineral oil (RoW)
**Uranium fuel element production, enriched 3.8 % for light water reactor**	market for cement, unspecified (RoW)<br>market for chromium (GLO)<br>market group for electricity, medium voltage (GLO)<br>market for heat, district or industrial, other than natural gas (RoW)<br>market for nuclear fuel factory (GLO)<br>Uranium, enriched 3.8% per separative work unit (calculated)<br>market for water, decarbonised (RoW)<br>market for inert waste, for final disposal (RoW)<br>market for low level radioactive waste (GLO)<br>market for wastewater, unpolluted (RoW)
**Nuclear fuel element production, for PWR, UO_2 3.8 % including MO_X**	market for MOX fuel element, for light water reactor (GLO)<br>Uranium fuel element production, enriched 3.8 % for light water reactor (calculated)

Supplementary Note 6

6 Annual Energy Investment Flows in the Energy Transition

The following figures illustrate the annual energy investment (EI) flows during the energy transition, categorised according to their intended use, i.e., the upstream supply chain for fuel production, operation, and investment. The systemwide energy investment flow (Supplementary Fig.19) is adopted as the denominator to estimate the systemwide EROI, while the annualised version of this flow is shown in Supplementary Fig.20. More explicitly, the systemwide energy investment flow is estimated using the cumulative capacities over time, considering the lifetime impact of the power plants. In contrast to this, the annual energy investment flow is estimated based on the invested annual gross capacities of the respective year and the energy requirements for operations are consider for the same period.

In the systemwide energy investment flow (Supplementary Fig. 19), the total energy requirement remains below 7% of the final energy consumption (FEC) for all scenarios during the energy transition. The upstream fuel production supply chain's share of the total energy investment is gradually decreasing and is being replaced by energy need for investments as the system moves towards defossilisation. In the IEA scenarios where fossil-fired power plants still account for a significant share of the energy mix, this change is inherently slower. It is noteworthy that the energy investments for the operational phase of the Teske/DLR scenarios are higher compared to the BPS scenarios, as bioenergy-related power plants are predominantly used instead of low-cost technologies (solar PV and wind power). On the other hand, a small increase in geothermal capacity has a decisive impact on the energy requirements for the investment phase, undeniably expresses the influence of the technology choice on the systemwide EROI.

The annual energy investment flow (Supplementary Fig. 20), which does not amortise the upfront energy investment over the plant lifetime after installation, shows the higher shares of energy requirement for both the operational and investment phases. Nevertheless, none of the scenarios reaches a FEC share of 16% during the energy transition. Similar results are observed for the systemwide energy investment flow. The energy need of the upstream supply chain for fuel production is substantially replaced by the energy necessity for the renewable energy investments, while the shares of the annual energy investment in the operational phase remain close to those of the systemwide energy investment flow. Consistent with the key findings in the paper, the shortening of the energy transition leads to a higher energy requirement for investments, which is particularly observed in the BPS-plus scenarios. On the other hand, the IEA scenarios do not show a significant change, as renewable energy capacity expansion is limited, and nuclear power is mostly preferred to be used instead fossil fuels. Yet, it does not lead to a significant reduction in the share of the energy requirement for the upstream supply chain for fuel production, which is the highest among the scenarios. In Teske/DLR scenarios, the annual EI flows for the given years do not exceed 11%, which gradually decreases with the slowdown in the expansion of bioenergy power plants, CSP ST and geothermal capacities.

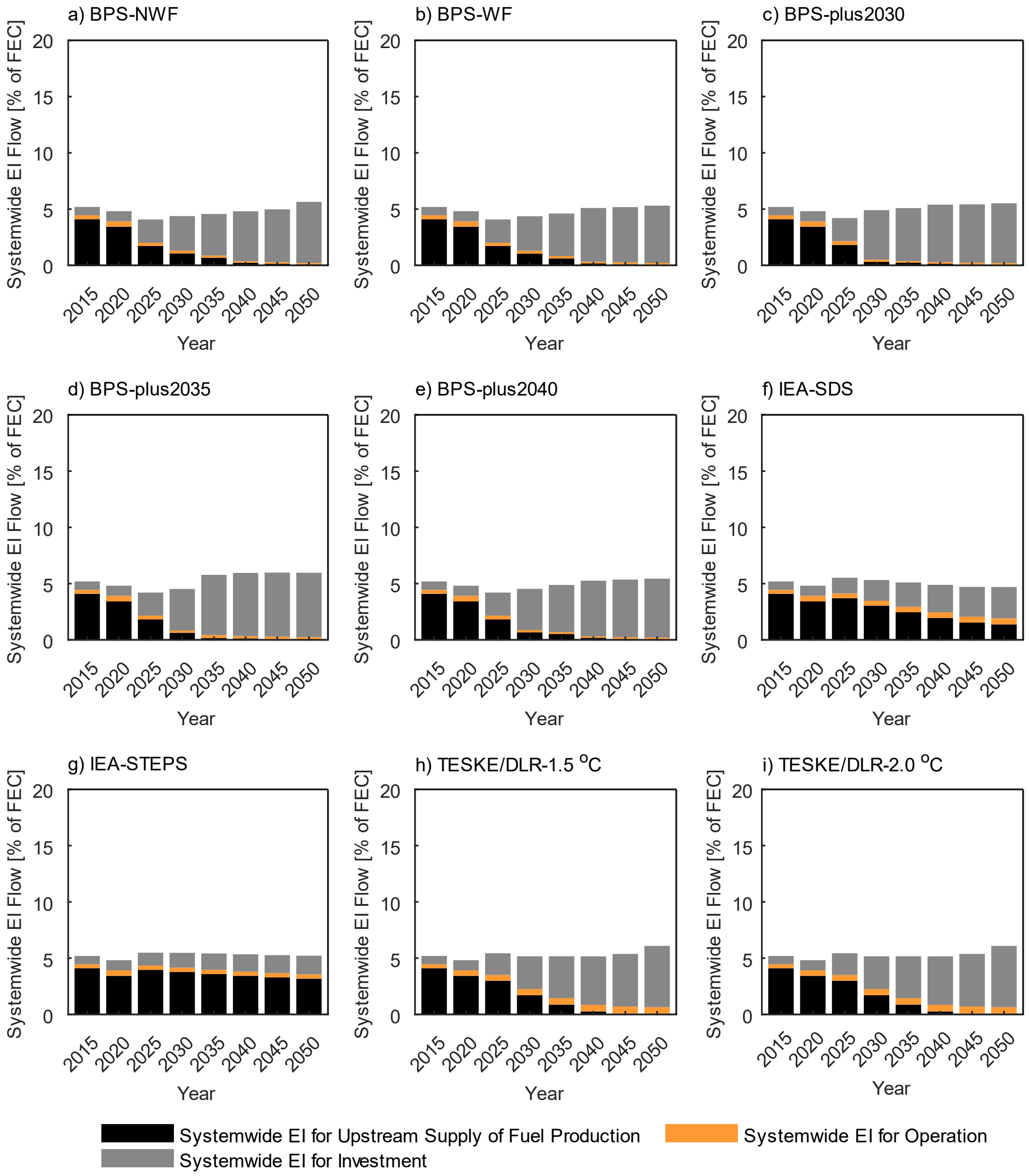

Supplementary Fig. 19. Systemwide energy investment flow for the global power system. The panels a, b, c, d, e, f show LUT scenarios, f and g show IEA scenarios, and h and i show Teske/DLR scenarios. EI refers to energy investments, and the annual EI for operation does not include structural materials. FEC represents the final energy consumption for the respective year.

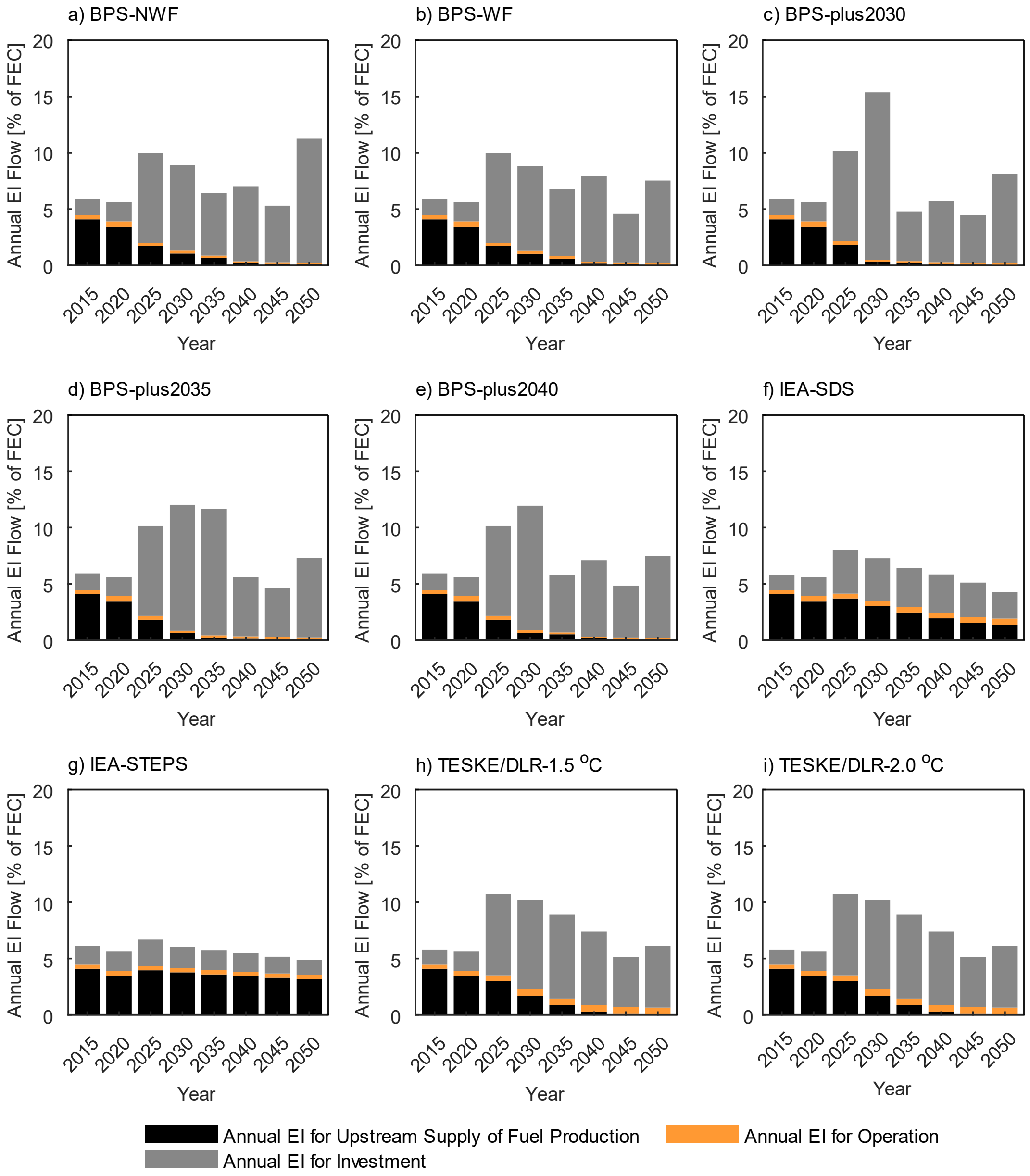

Supplementary Fig. 20. Annual energy investment flow for the global power system. The panels a, b, c, d, e, f show LUT scenarios, f and g show IEA scenarios, and h and i show Teske/DLR scenarios. EI refers to energy investments, and the annual EI for operation does not include structural materials. FEC represents the final energy consumption for the respective year.

References

1. Ram, M. *et al. Powerfuels in a Renewable Energy World- Global volumes, costs, and trading 2030 to 2050*. (2020).
2. Lopez, G. *et al.* Pathway to a fully sustainable energy system for Bolivia across power, heat, and transport sectors by 2050. *Journal of Cleaner Production* **293**, 126195 (2021).
3. Oyewo, A. S. *et al.* Just transition towards defossilised energy systems for developing economies: A case study of Ethiopia. *Renewable Energy* **176**, 346–365 (2021).
4. Bogdanov, D. *et al.* Low-cost renewable electricity as the key driver of the global energy transition towards sustainability. *Energy* **227**, 120467 (2021).
5. Sadiqa, A., Gulagi, A., Bogdanov, D., Caldera, U. & Breyer, C. Renewable energy in Pakistan: Paving the way towards a fully renewables-based energy system across the power, heat, transport and desalination sectors by 2050. *IET Renewable Power Generation* **16**, 177–197 (2022).
6. Bogdanov, D. *et al.* Radical transformation pathway towards sustainable electricity via evolutionary steps. *Nature Communications* **10**, 1–16 (2019).
7. Kuczenski, B. *et al.* LCA capability roadmap—product system model description and revision. *International Journal of Life Cycle Assessment* **23**, 1685–1692 (2018).
8. Weidema, B. P. *et al. Overview and methodology. Data quality guideline for the ecoinvent database version 3.* **Ecoinvent**, (2013).
9. Wernet, G. *et al.* The ecoinvent database version 3 (part I): overview and methodology. *International Journal of Life Cycle Assessment* **21**, 1218–1230 (2016).
10. Frischknecht, R. *et al. Overview and Methodology*. *Ecoinvent report No.1* (2007).
11. European Committee for Standardisation. CEN - EN 15804 - Sustainability of construction works - Environmental product declarations - Core rules for the product category of construction products. *Engineering360* (2012). Available at: https://standards.globalspec.com/std/14306124/EN 15804. (Accessed: 27th February 2022)
12. Ioannidou D. *et al. Documentation for the 'Allocation, cut-off, EN15804' system model*. (2021).
13. Harrison, G. P., Maclean, E. (Ned) J., Karamanlis, S. & Ochoa, L. F. Life cycle assessment of the transmission network in Great Britain. *Energy Policy* **38**, 3622–3631 (2010).
14. Omrany, H., Soebarto, V., Zuo, J. & Chang, R. A comprehensive framework for standardising system boundary definition in life cycle energy assessments. *Buildings* **11**, (2021).
15. Li, T., Zhang, H., Liu, Z., Ke, Q. & Alting, L. A system boundary identification method for life cycle assessment. *International Journal of Life Cycle Assessment* **19**, 646–660 (2014).
16. Klöpffer, W. & Grahl, B. *Life Cycle Assessment (LCA) - A Guide to Best Practice*. (Wiley-VCH, 2014).
17. International Organisation for Standardisation (ISO). ISO 14040:2006 - Environmental management - Life cycle assessment -Principles and framework. (2006). Available at: https://www.iso.org/standard/37456.html. (Accessed: 27th February 2022)
18. International Organization for Standardization (ISO). ISO 14044:2006 - Environmental management - Life cycle assessment - Requirements and guidelines. (2006). Available at: https://www.iso.org/standard/38498.html. (Accessed: 27th February 2022)
19. Hischier, R. *et al. Implementation of Life Cycle Impact Assessment Methods*. (2010).
20. European Committee for Standardisation. UNE EN 15643-2:2012 Sustainability of construction works - Assessment of buildings - Part 2: Framework for the assessment of environmental performance. *European Standards.* (2012). Available at: https://www.en-standard.eu/une-en-15643-2-2012-sustainability-of-construction-works-assessment-of-buildings-part-2-framework-for-the-assessment-of-environmental-performance/. (Accessed: 27th February 2022)

21. European Committee for Standardisation. BS EN 15978:2011 Sustainability of construction works. Assessment of environmental performance of buildings. Calculation method. *European Standards.* (2012). Available at: https://www.en-standard.eu/bs-en-15978-2011-sustainability-of-construction-works-assessment-of-environmental-performance-of-buildings-calculation-method/. (Accessed: 27th February 2022)

22. Wolfova, M. Analysing the cumulative energy demand of external bearing walls. *IOP Conference Series: Materials Science and Engineering* **867**, 8–13 (2020).

23. Wiesen, K. & Wirges, M. From cumulated energy demand to cumulated raw material demand: the material footprint as a sum parameter in life cycle assessment. *Energy, Sustainability and Society* **7**, (2017).

24. Raugei, M., Fullana-i-Palmer, P. & Fthenakis, V. The energy return on energy investment (EROI) of photovoltaics: Methodology and comparisons with fossil fuel life cycles. *Energy Policy* **45**, 576–582 (2012).

25. Murphy, D. J., Carbajales-Dale, M. & Moeller, D. Comparing apples to apples: Why the net energy analysis community needs to adopt the life-cycle analysis framework. *Energies* **9**, 1–16 (2016).

26. Murphy, D. J., Hall, C. A. S., Dale, M. & Cleveland, C. Order from chaos: A preliminary protocol for determining the EROI of fuels. *Sustainability* **3**, 1888–1907 (2011).

27. Arvesen, A. & Hertwich, E. G. More caution is needed when using life cycle assessment to determine energy return on investment (EROI). *Energy Policy* **76**, 1–6 (2015).

28. Carbajales-Dale, M., Raugei, M., Fthenakis, V. & Barnhart, C. Energy return on investment (EROI) of solar PV: An attempt at reconciliation [Point of View]. *Proceedings of the IEEE* **103**, 995–999 (2015).

29. Raugei, M. *et al.* Energy Return on Energy Invested (ERoEI) for photovoltaic solar systems in regions of moderate insolation: A comprehensive response. *Energy Policy* **102**, 377–384 (2017).

30. Sgouridis, S., Csala, D. & Bardi, U. The sower's way: Quantifying the narrowing net-energy pathways to a global energy transition. *Environmental Research Letters* **11**, (2016).

31. Solomon, A. A., Manjong, N. B. & Breyer, C. The necessity to standardise primary energy quality in achieving a meaningful quantification of related indicators. *Smart Energy* **12**, 100115 (2023).

32. Frischknecht, R., Wyss, F., Büsser Knöpfel, S., Lützkendorf, T. & Balouktsi, M. Cumulative energy demand in LCA: the energy harvested approach. *International Journal of Life Cycle Assessment* **20**, 957–969 (2015).

33. Kraan, O., Chappin, E., Kramer, G. J. & Nikolic, I. The influence of the energy transition on the significance of key energy metrics. *Renewable and Sustainable Energy Reviews* **111**, 215–223 (2019).

34. Frischknecht, R. *et al. Implementation of Life Cycle Impact Assessment Methods Data. ecoinvent Report No. 3, v2.0* (2007).

35. ecoinvent. ecoinvent database (V.3.7.1). (2020).

36. CoolConversion. 1 liter of oil engine in kg. *2021* Available at: https://coolconversion.com/density-volume-mass/--1--liter--of--oil-engine--in--kg. (Accessed: 22nd February 2022)

37. Cheminova A/S. Safety data sheet of glyphosate. (2018).

38. ASCO-TP. Concrete mix design. (2008). Available at: http://www.planete-tp.com/en/concrete-mix-design-a221.html. (Accessed: 23rd February 2022)

39. Perpiñan, O., Lorenzo, E., Castro, M. A. & Eyras, R. Energy payback time of grid connected PV systems: Comparison between tracking and fixed systems. *Progress in Photovoltaics: Research and Applications* **17**, 137–147 (2009).

40. Antonanzas, J., Arbeloa-Ibero, M. & Quinn, J. C. Comparative life cycle assessment of fixed and single axis tracking systems for photovoltaics. *Journal of Cleaner Production* **240**, 118016 (2019).

41. De Wild-Scholten, M. J. Energy payback time and carbon footprint of commercial photovoltaic

systems. *Solar Energy Materials and Solar Cells* **119**, 296–305 (2013).

42. Chen, Y. *et al.* From Laboratory to Production: Learning Models of Efficiency and Manufacturing Cost of Industrial Crystalline Silicon and Thin-Film Photovoltaic Technologies. *IEEE Journal of Photovoltaics* **8**, 1531–1538 (2018).

43. Fischer, M., Woodhouse, M., Herritsch, S. & Trube, J. *International Technology Roadmap for Photovoltaic (ITRPV)*. (2021).

44. Louwen, A., Van Sark, W. G. J. H. M., Faaij, A. P. C. & Schropp, R. E. I. Re-assessment of net energy production and greenhouse gas emissions avoidance after 40 years of photovoltaics development. *Nature Communications* **7**, 1–9 (2016).

45. Görig, M. & Breyer, C. Energy Learning Curves of PV Systems. *Environmental Progress & Sustainable Energy* **35**, 914–923 (2016).

46. REN21. *Renewables 2021 Global Status Report*. (2021).

47. SolarPower Europe. *Global Market Outlook for Solar Power 2021-2025.* (2021).

48. Patrizi, N. *et al.* Lifecycle environmental impact assessment of an overtopping wave energy converter embedded in breakwater systems. *Frontiers in Energy Research* **7**, 1–10 (2019).

49. Thomson, R. C., Chick, J. P. & Harrison, G. P. An LCA of the Pelamis wave energy converter. *International Journal of Life Cycle Assessment* **24**, 51–63 (2019).

50. Apolonia, M. & Simas, T. Life cycle assessment of an oscillating wave surge energy converter. *Journal of Marine Science and Engineering* **9**, 1–17 (2021).

51. Karan, H., Thomson, R. C. & Harrison, G. P. Full life cycle assessment of two surge wave energy converters. *Proceedings of the Institution of Mechanical Engineers, Part A: Journal of Power and Energy* **234**, 548–561 (2020).

52. Pennock, S., Vanegas Cantarero, M., Bloise Thomaz, T., Jeffrey, H. & Dickson, M. J. Life Cycle Assessment of a Point-Absorber Wave Energy Array. *SSRN Electronic Journal* (2021). doi:10.2139/ssrn.3870980

53. CorPower Completes World's Largest Wave Energy Test-Rig. *Off Grid Energy Independence* (2021). Available at: https://www.offgridenergyindependence.com/articles/24493/corpower-completes-worlds-largest-wave-energy-test-rig. (Accessed: 21st February 2022)

54. Singh, A., Christensen, T. & Panoutsou, C. Policy review for biomass value chains in the European bioeconomy. *Global Transitions* **3**, 13–42 (2021).

55. Nevzorova, T. & Kutcherov, V. Barriers to the wider implementation of biogas as a source of energy: A state-of-the-art review. *Energy Strategy Reviews* **26**, 100414 (2019).

56. Sivabalan, K., Hassan, S., Ya, H. & Pasupuleti, J. A review on the characteristic of biomass and classification of bioenergy through direct combustion and gasification as an alternative power supply. *Journal of Physics: Conference Series* **1831**, 0–22 (2021).

57. Vasilatos, C. *et al.* A Comparative Study of Selected Properties of Biomass and Coal Fuels from Greece. *Materials Proceedings* **5**, 108 (2022).

58. Adams, P. W. R. An assessment of UK bioenergy production, resource availability, biomass gasification and life cycle impacts. (University of Bath, 2011).

59. Adams, P. W. R. & McManus, M. C. Small-scale biomass gasification CHP utilisation in industry: Energy and environmental evaluation. *Sustainable Energy Technologies and Assessments* **6**, 129–140 (2014).

60. Abanades, S. *et al.* A critical review of biogas production and usage with legislations framework across the globe. *International Journal of Environmental Science and Technology* (2021). doi:10.1007/s13762-021-03301-6

61. Florio, C. *et al.* A life cycle assessment of biomethane production from waste feedstock through

different upgrading technologies. *Energies* **12**, 1–12 (2019).

62. Doka, G. *Life Cycle Inventories of Waste Treatment Services*. *Ecoinvent report No. 13* (2003).

63. Lausselet, C., Cherubini, F., del Alamo Serrano, G., Becidan, M. & Strømman, A. H. Life-cycle assessment of a Waste-to-Energy plant in central Norway: Current situation and effects of changes in waste fraction composition. *Waste Management* **58**, 191–201 (2016).

64. Ibikunle, R. A., Titiladunayo, I. F., Akinnuli, B. O., Dahunsi, S. O. & Olayanju, T. M. A. Estimation of power generation from municipal solid wastes: A case Study of Ilorin metropolis, Nigeria. *Energy Reports* **5**, 126–135 (2019).

65. IEA. *World Energy Outlook 2021*. (2021).

66. Carrara, S., Alves Dias, P., Plazzotta, B. & Pavel, C. *Raw materials demand for wind and solar PV technologies in the transition towards a decarbonised energy system*. *Jrc119941* (2020). doi:10.2760/160859

67. World Bank. *Concentrating Solar Power: Clean Power on Demand 24/7*. (2021). doi:10.1016/B978-0-08-102886-5.00019-0

68. National Renewable Energy Laboratory (NREL). Concentrating Solar Power Projects (SolarPACES). (2021). Available at: https://solarpaces.nrel.gov/. (Accessed: 26th February 2022)

69. Althaus, H. *et al. Life cycle inventories of chemicals. ecoinvent report No.8, v2.0.* (2007).

70. Koornneef, J., van Keulen, T., Faaij, A. & Turkenburg, W. Life cycle assessment of a pulverized coal power plant with post-combustion capture, transport and storage of CO2. *International Journal of Greenhouse Gas Control* **2**, 448–467 (2008).

71. Jiang, Y. LCA of Microgrid System : a Case Study at ‘ North - five Islands ’ of Changshan Archipelago , China. (KTH Royal Institute of Technology, 2019).

72. Carless, T. S., Griffin, W. M. & Fischbeck, P. S. The environmental competitiveness of small modular reactors: A life cycle study. *Energy* **114**, 84–99 (2016).

73. Ashley, S. F., Fenner, R. A., Nuttall, W. J. & Parks, G. T. Life-cycle impacts from novel thorium-uranium-fuelled nuclear energy systems. *Energy Conversion and Management* **101**, 136–150 (2015).

74. Ashley, S. F. *et al.* Fuel cycle modelling of open cycle thorium-fuelled nuclear energy systems. *Annals of Nuclear Energy* **69**, 314–330 (2014).

75. World Nuclear Association. Nuclear Power Reactors . (2021). Available at: https://world-nuclear.org/information-library/nuclear-fuel-cycle/nuclear-power-reactors/nuclear-power-reactors.aspx. (Accessed: 23rd February 2022)

76. World Nuclear Association. Plans for New Nuclear Reactors Worldwide . (2021). Available at: https://world-nuclear.org/information-library/current-and-future-generation/plans-for-new-reactors-worldwide.aspx. (Accessed: 23rd February 2022)

77. IAEA. *Energy, Electricity, and Nuclear Power Estimates for the Period up to 2050*. (2021).

78. IAEA. *Nuclear Power Reactors in the World*. (2021).

79. Arshad, F. *et al.* Life Cycle Assessment of Lithium-ion Batteries: A Critical Review. *Resources, Conservation and Recycling* **180**, (2022).

80. Chen, T. *et al.* Applications of Lithium-Ion Batteries in Grid-Scale Energy Storage Systems. *Transactions of Tianjin University* **26**, 208–217 (2020).

81. Westlake, B. *Recycling and Disposal of Battery-Based Grid Energy Storage Systems: A Preliminary Investigation*. *Electric Power Research Institute (EPRI)* (2017).

82. Pellow, M. A., Ambrose, H., Mulvaney, D., Betita, R. & Shaw, S. Research gaps in environmental life cycle assessments of lithium ion batteries for grid-scale stationary energy storage systems: End-of-life options and other issues. *Sustainable Materials and Technologies* **23**, e00120 (2020).

83. Notter, D. A. *et al.* Contribution of Li-ion Batteries to the Environmental Impact of Electric Vehicles.

Environmental Science and Technology **44**, 6550–6556 (2010).

84. Xu, C. *et al.* Future material demand for automotive lithium-based batteries. *Communications Materials* **1**, (2020).

85. Hsieh, I. Y. L., Pan, M. S., Chiang, Y. M. & Green, W. H. Learning only buys you so much: Practical limits on battery price reduction. *Applied Energy* **239**, 218–224 (2019).

86. Greim, P., Solomon, A. A. & Breyer, C. Assessment of lithium criticality in the global energy transition and addressing policy gaps in transportation. *Nature Communications* **11**, 1–11 (2020).

87. Kapila, S., Oni, A. O., Gemechu, E. D. & Kumar, A. Development of net energy ratios and life cycle greenhouse gas emissions of large-scale mechanical energy storage systems. *Energy* **170**, 592–603 (2019).

88. Alves, B. World electricity generation share by energy source. *Statista* (2021). Available at: https://www.statista.com/statistics/269811/world-electricity-production-by-energy-source/. (Accessed: 23rd February 2022)

89. Spielmann, M., Ruiz, S. & Zah, R. *Analyse der Umwelt-Hotspots von Strombasierten Treibstoffen - Finaler Bericht*. (2015).

90. Wettstein, S., Itten, R. & Stucki, M. *Life Cycle Assessment of Renewable Methane for Transport and Mobility*. (2018).

91. Blanco, H. *et al.* Life cycle assessment integration into energy system models: An application for Power-to-Methane in the EU. *Applied Energy* **259**, 114160 (2020).

92. Bertuccioli, L. *et al. Study on development of water electrolysis in the EU. Final Report in Fuel Cells and Hydrogen, Joint Undertaking* **1**, (2014).

93. ISINNOVA. Final Report Summary - NEEDS (New Energy Externalities Development for Sustainability) |. *EU CORDIS Website* (2012). Available at: https://cordis.europa.eu/project/id/502687/reporting. (Accessed: 24th February 2022)

94. National Research Council and National Academy of Engineering. *The Hydrogen Economy: Opportunities, Costs, Barriers, and R&D Needs*. (National Academies Press, 2004). doi:10.17226/10922

95. Böhm, H., Goers, S. & Zauner, A. Estimating future costs of power-to-gas – a component-based approach for technological learning. *International Journal of Hydrogen Energy* **44**, 30789–30805 (2019).

96. Fasihi, M., Efimova, O. & Breyer, C. Techno-economic assessment of CO2 direct air capture plants. *Journal of Cleaner Production* **224**, 957–980 (2019).

97. Marchese, M., Buffo, G., Santarelli, M. & Lanzini, A. CO2from direct air capture as carbon feedstock for Fischer-Tropsch chemicals and fuels: Energy and economic analysis. *Journal of CO2 Utilization* **46**, 101487 (2021).

98. Madhu, K., Pauliuk, S., Dhathri, S. & Creutzig, F. Understanding environmental trade-offs and resource demand of direct air capture technologies through comparative life-cycle assessment. *Nature Energy* **6**, 1035–1044 (2021).

99. Deutz, S. & Bardow, A. Life-cycle assessment of an industrial direct air capture process based on temperature–vacuum swing adsorption. *Nature Energy* **6**, 203–213 (2021).

100. Götz, M. *et al.* Renewable Power-to-Gas: A technological and economic review. *Renewable Energy* **85**, 1371–1390 (2016).

101. Zhang, X., Witte, J., Schildhauer, T. & Bauer, C. Life cycle assessment of power-to-gas with biogas as the carbon source. *Sustainable Energy and Fuels* **4**, 1427–1436 (2020).

102. Swedish Gas Technology Centre. *Basic Data on Biogas*. *Medical Care Research and Review* **2nd Editio**, (2012).

103. BP p.l.c. *Stratistical Review of World Energy*. **69th editi**, (2020).

104. Caglayan, D. G. *et al.* Technical potential of salt caverns for hydrogen storage in Europe. *International Journal of Hydrogen Energy* **45**, 6793–6805 (2020).

105. The Engineering Toolbox. Fuels - Higher and Lower Calorific Values. Available at: https://www.engineeringtoolbox.com/fuels-higher-calorific-values-d_169.html. (Accessed: 22nd November 2022)

106. Lopez, G., Farfan, J. & Breyer, C. Trends in the global steel industry: Evolutionary projections and defossilisation pathways through power-to-steel. *Journal of Cleaner Production* **375**, 134182 (2022).

107. Delannoy, L., Longaretti, P. Y., Murphy, D. J. & Prados, E. Peak oil and the low-carbon energy transition: A net-energy perspective. *Applied Energy* **304**, 117843 (2021).

108. Delannoy, L., Longaretti, P. Y., Murphy, D. J. & Prados, E. Assessing global long-term EROI of gas: A net-energy perspective on the energy transition. *Energies* **14**, 1–16 (2021).

109. Rye, C. D. & Jackson, T. A review of EROEI-dynamics energy-transition models. *Energy Policy* **122**, 260–272 (2018).
